# Harnessing Baseline Radiomic Features in Early-Stage NSCLC: What Role in Clinical Outcome Modeling for SBRT Candidates?

**DOI:** 10.3390/cancers17050908

**Published:** 2025-03-06

**Authors:** Stefania Volpe, Maria Giulia Vincini, Mattia Zaffaroni, Aurora Gaeta, Sara Raimondi, Gaia Piperno, Jessica Franzetti, Francesca Colombo, Anna Maria Camarda, Federico Mastroleo, Francesca Botta, Lorenzo Spaggiari, Sara Gandini, Matthias Guckenberger, Roberto Orecchia, Monica Casiraghi, Barbara Alicja Jereczek-Fossa

**Affiliations:** 1 Division of Radiation Oncology, IEO, European Institute of Oncology, IRCCS, 20141 Milan, Italy; stefania.volpe@ieo.it (S.V.); gaia.piperno@ieo.it (G.P.); jessica.franzetti@cnao.it (J.F.); francesca.colombo@cnao.it (F.C.); annamaria.camarda@cnao.it (A.M.C.); federico.mastroleo@ieo.it (F.M.); barbara.jereczek@ieo.it (B.A.J.-F.); 2 Department of Oncology and Hemato-Oncology, University of Milan, 20122 Milan, Italy; lorenzo.spaggiari@ieo.it (L.S.); monica.casiraghi@ieo.it (M.C.); 3 Molecular and Pharmaco-Epidemiology Unit, Department of Experimental Oncology, IEO, European Institute of Oncology, IRCCS, 20141 Milan, Italy; aurora.gaeta@ieo.it (A.G.); sara_raimondi@hotmail.com (S.R.); 4 Department of Statistics and Quantitative Methods, Università degli Studi di Milano-Bicocca, 20125 Milan, Italy; 5 Medical Physics Unit, IEO, European Institute of Oncology, IRCCS, Via Ripamonti 435, 20141 Milan, Italy; francesca.botta@asst-settelaghi.it; 6 Division of Thoracic Surgery, IEO, European Institute of Oncology, IRCCS, 20141 Milan, Italy; 7 Department of Experimental Oncology, IEO, European Institute of Oncology, IRCCS, 20141 Milan, Italy; sara.gandini@ieo.it; 8 Department of Radiation Oncology, University Hospital Zurich, University of Zurich, 8091 Zurich, Switzerland; matthias.guckenberger@usz.ch; 9 Scientific Directorate, IEO, European Institute of Oncology, IRCCS, 20141 Milan, Italy; roberto.orecchia@ieo.it

**Keywords:** radiomics, outcome modeling, early-stage non-small cell lung cancer, SBRT

## Abstract

**Aim:** An Early-Stage Non-Small Cell Lung Cancer (ES-NSCLC) patient candidate for stereotactic body radiotherapy (SBRT) may start their treatment without a histopathological assessment, due to relevant comorbidities. The aim of this study is twofold: (i) build prognostic models to test the association between CT-derived radiomic features (RFs) and the outcomes of interest (overall survival (OS), progression-free survival (PFS) and loco-regional progression-free survival (LRPFS)); (ii) quantify whether the combination of clinical and radiomic descriptors yields better prediction than clinical descriptors alone in prognostic modeling for ES-NSCLC patients treated with SBRT. **Methods:** Simulation CT scans of ES-NSCLC patients treated with curative-intent SBRT at the European Institute of Oncology (IEO), Istituto di Ricovero e Cura a Carattere Scientifico (IRCCS), Milan, Italy between 2013 and 2023 were retrospectively retrieved. PyRadiomics v3.0.1 was used for image preprocessing and subsequent RFs extraction and selection. A radiomic score was calculated for each patient, and three prognostic models (clinical model, radiomic model, clinical-radiomic model) for each survival endpoint were built. Relative performances were compared using the C-index. All analyses were considered statistically significant if *p* < 0.05. The statistical analyses were performed using R Software version 4.1. **Results:** A total of 100 patients met the inclusion criteria. Median age at diagnosis was 76 (IQR: 70–82) years, with a median Charlson Comorbidity Index (CCI) of 7 (IQR: 6–8). At the last available follow-up, 76 patients were free of disease, 17 were alive with disease, and 7 were deceased. Considering relapses, progression of any kind was diagnosed in 31 cases. Regarding model performances, the radiomic score allowed for excellent prognostic discrimination for all the considered endpoints. Of note, the use of RFs alone proved to be more informative than clinical characteristics alone for the prediction of both OS and LRPFS, but not for PFS, for which the individual predictive performances slightly favored the clinical model. **Conclusion:** The use of RFs for outcome prediction in this clinical setting is promising, and results seem to be rather consistent across studies, despite some methodological differences that should be acknowledged. Further studies are being planned in our group to externally validate these findings, and to better determine the potential of RFs as non-invasive and reproducible biomarkers in ES-NSCLC.

## Simple Summary

This study aims to assess whether non-invasive radiomic features (RFs) derived from CT scans could improve survival predictions for Early-Stage Non-Small Cell Lung Cancer (ES-NSCLC) patients treated with stereotactic body radiotherapy (SBRT). Three prognostic models were built: clinical, radiomic, and combined clinical-radiomic, and their predictive accuracy for overall survival (OS), progression-free survival (PFS), and loco-regional progression-free survival (LRPFS) were compared using the C-index. Data from 100 patients were analyzed. Results showed that the radiomic model provided superior prediction for OS and LRPFS compared to clinical factors alone, though clinical models slightly outperformed RFs for PFS. The findings support the potential of RFs as non-invasive biomarkers for outcome prediction in ES-NSCLC patients. Future studies are planned to validate these results and further explore RFs’ utility in clinical practice.

## 1. Introduction

With almost 2.5 million cases in 2022 [1], lung cancer is ranked as the third most commonly diagnosed cancer, contributing to over 20% of cancer-related deaths. Of these, 85% of cases are classified as non-small cell lung cancer (NSCLC) [2]. In early-stage NSCLC (ES-NSCLC), current guidelines indicate surgery as the treatment of choice, while stereotactic body radiotherapy (SBRT) serves as a secondary option for those with contraindications to major surgical intervention and/or general anesthesia [3]. This generally results in a negative selection of SBRT, for whom even a bioptic procedure may be burdened with potentially severe complications, including pleural effusion, infections, and anesthesia-related side effects.

In this regard, radiomics-derived biomarkers are particularly promising, thanks to image availability, relative ease of extraction, repeatability over time, and low implementation costs [4,5]. Ideally, a reliable radiomic biomarker could be integrated with clinical data, including but not limited to the patient’s age, gender, performance status, and baseline symptoms, in order to refine prognostic stratification. Ten years after the first publication on radiomics, lung cancer has indeed emerged as one of the most relevant topics, ranking among the 50 most frequent keywords, as noted in a recent bibliometric analysis [6].

However, as of early 2024, no radiomic signature has been validated for clinical use in this setting. This has been underlined in a metanalysis by Kothari et al. [7], who showed that the overall ability to predict overall survival is largely unsatisfactory, with a C-index random effect estimate of 0.57 (95% CI 0.53–0.62). Such a modest performance can be explained by multiple factors affecting the radiomic workflow at multiple levels, from study design to results reporting. These apply also to the comparatively low number of original investigations on computed tomography (CT)-based radiomics for outcome prediction in ES-NSCLC patients treated with SBRT. Among the most common biases, it is worth mentioning the inclusion of pulmonary oligo-metastases [8], limited sample size [9], heterogeneous acquisition parameters [10,11], and the use of a non-standardized ontology [11]. Additionally, specific methodological aspects have often been neglected in historical publications, including the potential informative content of 4-dimensional CT simulation scans or the impact of image filtering.

Moving from such a background, the present study aims to investigate the oncological outcomes of a homogenous cohort of ES-NSCLC patients treated at a single tertiary care cancer center, to assess the impact of the addition of radiomic features (RFs) extracted from lung nodules contoured on original and filtered non-contrast enhanced CT simulation scans. In detail, the specific aims are as follows:

Build prognostic models to test the association between CT-derived RFs and the following outcomes of interest: overall survival (OS), progression-free survival (PFS), and loco-regional progression-free survival (LRPFS).

Quantify whether the combination of clinical and radiomic descriptors yields better prediction than clinical descriptors alone in prognostic modeling for ES-NSCLC patients treated with SBRT.

## 2. Patients and Methods

### 2.1. Clinical Dataset

Patients treated at the European Institute of Oncology (Istituto Europeo di Oncologia, IEO) IRCCS, Milan, Italy between 2013 and 2023 were retrospectively retrieved after formal approval of the local Ethical Committee (UID 4219, approved on 14 July 2023).

Inclusion criteria were as follows: (1) histological and/or radiological diagnosis of NSCLC, (2) early-stage disease, classified according to the American Joint Committee on Cancer (AJCC) tumor, node, metastasis (TNM), 8th edition (e.g., clinical stage I-II), (3) curative-intent SBRT administered at the IEO IRCSS to a minimum biologically effective dose (BED) of 100 Gy, (4) availability of four-dimensional simulation CT (4D-CT) Digital Imaging and Communications in Medicine (DICOM) files acquired, (5) minimum follow-up of at least 6 months since diagnosis, and (6) availability of the written informed consent for the anonymous use of data for research purposes, which was verified for each patient. Conversely, patients with recurrent NSCLC tumors and/or those who had received prior irradiation in the thoracic region were not considered. The diagnosis of other malignancies in the three years preceding the diagnosis of ES-NSCLC, except for skin neoplasms, also served as an exclusion criterion.

Notably, all prescription doses were recalculated as BEDs, assuming an α/β ratio of 10 Gy for tumor control [12]. This choice was made to favor interpretability and to ensure uniform input data for the analyses. For completeness, the number of fractions, dose/fraction, and nominal total prescription dose data were also recorded.

Acquisition parameters were consistent for all patients: helical acquisition mode, slice thickness of 2.5 mm, kiloVolt peak of 120 kV, and an x-ray tube current of 200 mA. All simulation CT scans were acquired with the same machine, a GE (General Electric Company, Boston, MA, USA) Medical System Optima CT 580 scanner, without contrast medium injection, and were acquired under free-breathing conditions.

### 2.2. Tumor Delineation and RF Extraction

For tumor delineation and RFs extraction, the average reconstruction of each 4D-acquired CT scan was considered. To avoid inter-observer variability, all lung nodule segmentations (i.e., the GTV) were performed by a single Radiation Oncologist (SV). All radiation therapy (RT) structures and the average 4D-CT scan, both in DICOM format, were then exported from Raystation version 11A [RaySearch Laboratories] to PyRadiomics v3.0.1 for image preprocessing and subsequent RF extraction. RFs were extracted from lung nodules contoured on original and filtered non-contrast enhanced reconstructed average scans.

All available filters were applied. Specifically, all nine permutations of a high (H) and low (L) pass filter in the XYZ directions were calculated for the wavelet filter (e.g., HHH, HHL, LLL); similarly, subcategories of the lbp-3D image type were computed as well (namely, the kurtosis map- lbp-3D-k, and two additional harmonics, lbp-3D-m1 and lbp-3D-m2). Finally, 1, 2, and 5 were chosen as sigma values for the LoG image type. All the remaining parameters, including bin width, were left as default values. Since CT intensities were homogenous within the dataset, no intensity normalization or image resampling were needed.

### 2.3. Statistical Analysis

Frequencies and medians (with first and third quartiles) were used to describe categorical and continuous variables, respectively. Oncological outcomes considered for the analyses were:

i. OS, defined as the time length from diagnosis to death from any cause or last contact at follow-up;
ii. PFS, defined as the time length from diagnosis to any disease progression or death from any cause or to lost contact at follow-up;
iii. LRPFS, defined as the time length from diagnosis to local disease progression or death.

A brief explanation of the main clinical variables (i.e., those included for model building) is provided in Supplementary Materials Table S1.

### 2.4. Feature Selection and Radiomic Score Calculation

Features with near-zero variance and high correlation (Spearman ρ > 0.95) were excluded. The remaining RFs were clustered by an iterative clustering algorithm, grouping features when Spearman ρ > 0.75. Within each cluster, the RF with the strongest association with each outcome of interest was retained (i.e., the RF with the lowest *p*-value from the Cox Proportional Hazard (PH) univariate regression model). The procedure was iterated until the Spearman ρ was always <0.75 among all the selected RF. For each RF, a coefficient was obtained by the Multivariable Cox—LASSO Regression Model, where a higher coefficient value indicates a greater contribution to the prediction. The radiomic score for each patient was then calculated by summing the RF, each multiplied by its corresponding coefficient.

### 2.5. Comparison of Prognostic Models

The study evaluated three prognostic models for each survival endpoint. they were the clinical model—containing only clinical information; radiomic model—containing only the radiomic score; and the clinical-radiomic model—containing both clinical information and the radiomic score.

Univariate Cox PH regression models were used to test associations of clinical variables and radiomic score with the endpoints of interest; cut-off points for clinical continuous variables were chosen according to their clinical significance or, if unknown, by using the median value. Variables with *p* ≤ 0.10 in univariate analysis were included in multivariable analysis and retained if the *p*-value was confirmed as ≤0.10. In order to avoid collinearity, only one of the different significantly correlated variables was included in the multivariable model: the one with the lowest *p*-value in the univariate analysis. Risk estimates were quantified by the Hazard Ratio (HR) and 95% confidence intervals (CI). A log-rank test was used to compare survival curves by significant variable strata. For the radiomic score, the median was used to dichotomize the variable into high and low radiomic scores.

Clinical, radiomic, and clinical-radiomic models were compared using the C-index, a goodness of fit measure for binary outcomes ranging from a very poor predictive model (0.5) to a hypothetically perfect predictive model (1.0). For each clinical-radiomic model, an internal 3-fold cross-validation was implemented for each clinical outcome and repeated 500 times with different random seeds to ensure the robustness and stability of the results. The training and test sets followed a 2:1 fold distribution, ensuring that each fold of the 3-fold cross-validation trained and validated the model on different subsets of data. This approach was chosen based on the number of events and variables to reduce the risk of overfitting and provide a balanced evaluation of model performance. The median and interquartile range (IQR) of the C-Index estimated across all 500 iterations were reported.

All analyses were considered statistically significant if *p* < 0.05. The statistical analyses were performed using R Software version 4.1.

## 3. Results

### 3.1. Patient- and Tumor Characteristics

A total of one hundred patients treated between September 2013 and July 2023 met the inclusion criteria and were included in the analysis. The median age at diagnosis was 76 (IQR: 70–82) years, with nearly all patients (n = 92) presenting with at least one comorbidity; median Charlson Comorbidity Index (CCI) was 7 (IQR: 6–8). The most frequently represented stages were IA2 (n = 46) and IB (n = 21). Overall, 54 patients underwent histopathological assessment, which resulted in the diagnosis of adenocarcinoma in 41 cases, and of squamous cell carcinoma in the remaining 13 cases. Median overall treatment time (OTT) was 5 days. The median delivered BED was 124.8 (IQR: 112.5–151.2) Gy. All patient- and tumor-related baseline characteristics are provided in Table 1. Further details on fractionation schedules are provided in Supplementary Materials Table S2.

At the last available follow-up, 76 patients were free of disease, 17 were alive with disease, and 7 were deceased. Considering relapses, progression of any kind was diagnosed in 31 cases. Further details on the follow-up, sites of relapse, and treatment at progression are provided in Supplementary Materials Table S3.

### 3.2. Overall Survival

Median follow-up time was 2.04 (IQR: 1.23–3.84) years. The univariate analysis proved the following clinical variables to be statistically significant: median BED (*p* = 0.022, HR: 0.08, 95% CI 0.01–0.69), CCI (*p* = 0.018, HR: 1.74, 95% CI 1.10–2.76), and OTT (*p* = 0.015, HR: 1.40, 95% CI 1.07–1.83). However, only the median BED maintained statistical significance at multivariable analysis, and allowed for excellent prognostic stratification, with doses <124.8 Gy being associated with a detrimental prognostic effect (*p* = 0.004). The Kaplan-Meier curve is provided in Supplementary Materials Figure S1.

**Figure 1:**
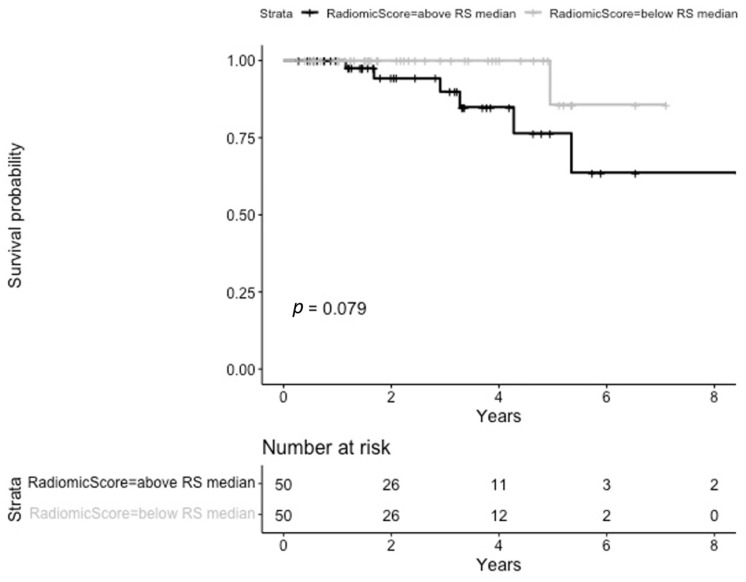
Kaplan-Meier curves stratified according to the radiomic score for overall survival.

In total, 1967 features were extracted from each lung nodule segmentation. A list of all the extracted radiomic features in shown in Supplementary Materials Table S4 After feature selection, three RFs were selected for the Radiomic Model, all from the gray level matrix filter category, namely GLRLM_RunLenghtNonUniformity (from the exponential- filtered image), GLCM_ClusterProminence (from the square root-filtered image), and GLDM_LargeDependenceHighGrayLevelEmphasis (from the wavelet LHL-filtered image). Median RS was 0.51 (IQR 0.40–1.07). A borderline difference was observed between high- and low- radiomic score subgroups (log-rank test *p* = 0.079), as shown in Figure 1.

### 3.3. Progression-Free Survival

The median PFS was 4.27 95% CI [3.09; NA] years. The univariate analyses showed statistical significance for the following variables: Forced Expiratory Volume (FEV)1% (*p* = 0.05, HR: 0.99, 95% CI 0.97–1.00), shape (*p* = 0.022, HR: 0.23, 95% CI 0.07–0.81 for oval shapes), and margins (*p* = 0.017, HR: 0.33 for irregular margins, 95% CI 0.14–0.82). Of these, at the multivariable analysis, only FEV1% and shape retained significance, and were therefore included in the clinical and clinico-radiomic models.

**Figure 2:**
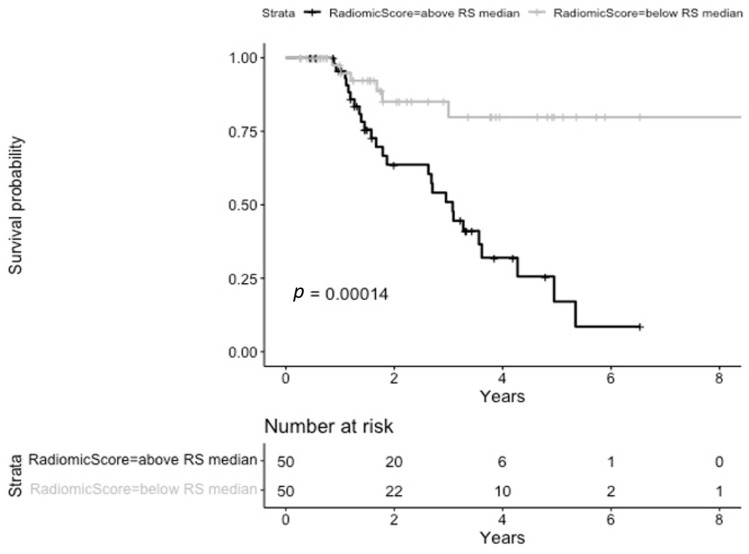
Kaplan-Meier curves stratified according to the radiomics score for progression-free survival.

Considering radiomic-related information, seven features were associated with PFS, all from the gray level matrix filter category, as follows: GLSZM_GrayLevelVariance (from the original image), GLSZM_SmallAreaEmphasis (from the lbp-2D- filtered image), GLRLM_RunLengthNonUniformityNormalized (from the lbp-3D-k- filtered image), GLCM_Correlation (from the log-sigma-0-59375-mm-3D- filtered image), GLDM_SmallDependenceHighGrayLevelEmphasis (from the logarithm- filtered image), GLSZM_SizeZoneNonUniformityNormalized (from the wavelet-LHL- filtered image), and GLCM_Correlation (from the wavelet-HLL- filtered image). Median RS was 1.45 (IQR 1.35–1.54). The median cut-off was successful in stratifying patients according to their risk of progression, with patients with a higher score being at higher risk (log-rank test *p* = 0.00014), as shown in Figure 2.

### 3.4. Loco Regional Progression-Free Survival

For this endpoint, the median available follow-up was 4.27 95% CI [3.09; NA] years. At the univariate analysis, the following clinical variables were statistically significant: Diffusing Capacity Of The Lungs For Carbon Monoxide (DLCO) (*p* = 0.014, HR: 0.96, 95% CI 0.93–0.99), and FEV1% (*p* = 0.019, HR: 0.97, 95% CI 0.95–1.00), with only FEV1% maintaining statistical significance at the multivariable analysis.

**Figure 3:**
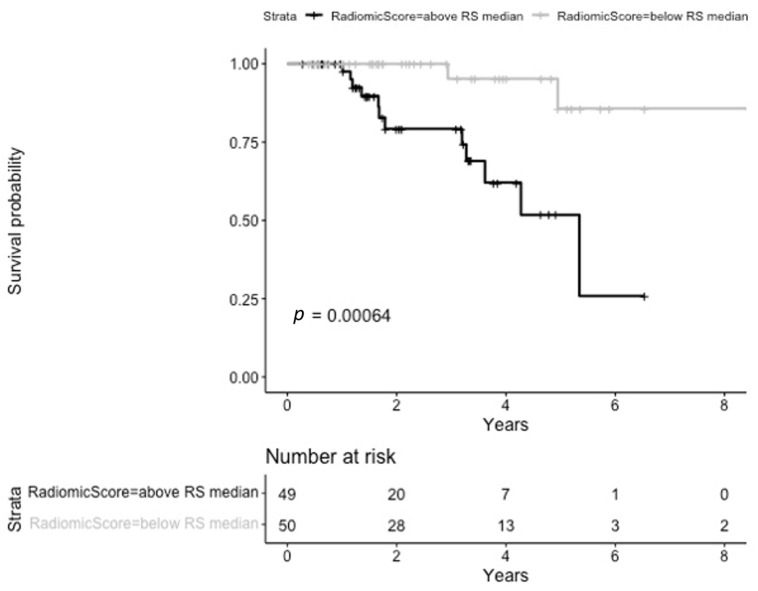
Kaplan-Meier curves stratified according to the radiomics score for local progression-free survival.

The radiomic model selected five RFs, namely the original_shape_Elongation (from the original image), firstorder_Kurtosis (from the lbp-3D-m1- filtered image), GLSZM_SizeZoneNonUniformity (from the log-sigma-2-96875-mm-3D- filtered image), GLCM_Contrast (from the logarithm- filtered image) and GLSZM_GrayLevelVariance (from the square-root-filtered image). Median RS was 1.28 (IQR 1.06–1.47). Patients whose radiomic score was below the median had better local disease control than those whose radiomic score was higher than the median (log-rank test *p* = 0.00064); this information is provided in Figure 3.

Results of the multivariable analyses for the clinical, radiomic, and clinico-radiomic model are reported in Table 2, while Table 3 provides an overview of model performances.

## 4. Discussion

Results of the present study show that the integration of baseline CT-derived features yields an improvement in the prognostic stratification of ES-NSCLC candidates to curative-intent SBRT. Specifically, we could demonstrate that the radiomic score allowed for excellent prognostic discrimination for all the considered endpoints, and that the hybrid model consistently yielded the best predictive ability. Of note, the use of RFs alone proved to be more informative than clinical characteristics alone for the prediction of both OS and LRPFS, but not for PFS, for which the individual predictive performances slightly favored the clinical model.

Considering clinical variables, FEV1% was retained as a significant variable in both the clinical and clinico-radiomic model for PFS and LRPFS, but not for OS. To date, evidence on the prognostic role of respiratory function in lung cancer is scarce, and largely contradictory [13,14,15,16]. However, a recent publication by Zhai et al. [15] on a large cohort from the Boston Lung Cancer Study (n = 2805) has investigated the association between spirometry values and OS, identifying that lower quartiles of actual and percent FEV1% as well as forced vital capacity at diagnosis were significantly associated with worse OS [15]. Of note, none of these patients were SBRT candidates, and the study examines data from all disease stages. Hence, it is not clear whether these findings may be translated to curative-intent SBRT for early-stages. Historically, spirometry parameters have not been regarded as prognosticators, but rather as clinical parameters to orient clinicians’ decisions towards indicating or not indicating an extremely hypofractionated treatment to the lungs. To date, even severe COPD is not considered an absolute contraindication to SBRT, as studies have shown that such treatment is not associated with either reduced post-treatment pulmonary function or with a decreased quality of life, even in fragile patients [13,17,18].

Our results highlight a relevant unmet need and should encourage dedicated research. It should also be noted that the magnitude of our findings is limited by several factors, including the relatively small sample size and, more importantly, the presence of missing data. For instance, spirometry parameters could be retrieved in 84% of cases, and other clinically relevant data were not systematically available. Of these, it is worth mentioning that one of these is the number of pack/years, a standardized measurement of smoking habits, with a well-recognized independent prognostic relevance in patients with irradiated lungs [19].

Considering RFs, the LASSO mostly selected features of the higher-order statistics category (13/15, ~87%). This class includes RFs describing the spatial relationship among voxels in a volume of interest, such as the GTV. As an example, the measurement of the variability of gray-level intensity values in an image corresponds to the Gray Level Non-Uniformity Normalized (GLNN) features, which belong to the GLSZM category. The frequent inclusion of high-order features in the models suggests a potential association between tumor density and oncological outcomes, both in terms of OS and disease progression (namely, PFS and LRPFS).

Other than the consistency of this finding within this cohort, rather comparable results were reported by Sawayanagi et al. [20], who proposed clinico-radiomic prediction models in the setting of ES-NSCLC patients treated with SBRT. As in this series, the authors used PyRadiomics to extract RFs from simulation CTs acquired at free-breathing, though some differences in the acquisition protocol must be acknowledged. Namely, slice thickness was inferior (2 mm vs. 2.5 mm) and the tube current was higher (350 mA vs. 200 mA), while the tube voltage was the same as in our series (i.e., 120 kV). Of note, Sawayanagi et al. [20] did not enable all filtering modalities, but focused solely on the permutations of the wavelet. While the authors did not identify any radiomic signature predictive of PFS and LRPFS, they could describe an association between one high-order feature and OS, which was confirmed at external validation. Specifically, it is the “LargeAreaEmphasis_LHH”, a feature of the GLSZM category describing the “measure of the distribution of large area size zones, with a greater value indicative of more large size zones and more coarse textures”, as per the IBSI definition [21]. Specifically, this RF alone allowed for efficient prognostic stratification in the validation cohort (n = 108), with a significant difference in OS (*p* = 0.044, 5-year OS of 70.7% vs. 50.3% in the group with low and high values of the LargeAreaEmphasis_LHH feature, respectively). Therefore, in plainer language, lesions with a finer and more regular texture seem to be associated with better outcomes, at least in terms of survival.

While in this series, other higher-order RFs had been retained as associated with OS (i.e., GLRLM, GLCM and GLDM), features of the GLSZM category are present in the models for PFS and LRPFS, with 3/7 and 1/5 elements, respectively. In particular, the “SmallAreaEmphasis_lbp-2D”, included in the radiomic model for PFS, indicates “the distribution of small size zones, with a greater value indicative of smaller size zones and more fine textures” [21], and therefore seems to suggest a meaning similar to the “LargeAreaEmphasis_LHH” identified by Sawayanagi et al. Interestingly, the “SizeZoneNonUniformityNormalized_LHL” and its non-normalized equivalent, extracted from the log-sigma filtered CT, were associated with disease progression as well. Specifically, they indicated the (normalized) “variability of size zone volumes in the image, with a lower value indicating more homogeneity in size zone volumes”.

The fact that these results imply at least some informative signal is further confirmed by earlier findings by Lafata et al. [22], who retrieved data from a cohort of 70 stage I NSCLC SBRT candidates. In this case, two different scanners were used for the acquisition of free-breathing simulation CTs, albeit under the same calibration modalities. Another potential limitation is the use of in-house developed software, whose compliance with the IBSI recommendations is not explicitly declared. However, the authors extracted 43 RFs, sorted into four classes, namely intensity, fine texture, morphological, and coarse texture. The latter included features with a similar meaning as the ones discussed above, such as the “Gray Level Non-Uniformity” and the “Short Run Low Gray Level Emphasis”. In this series as well, a coarse texture was associated with higher rates of local recurrence, as indicated by the “Long-Run-High-Gray-Level-Emphasis” (*p* = 0.048). Additionally, the “Homogeneity2” feature, indicating denser lesions, showed a negative association with prognosis (*p* = 0.022), which was true also in our series. Specifically, two RFs indicating texture homogeneity were associated with either OS (i.e., the “RunLenghtNonUniformity_exponential”) or PFS (i.e., the “SmallDependenceHighGrayLevelEmphasis”).

On a more general level, it is possible to observe that all but two of the LASSO-selected RFs (~87%) were derived from filtered images. This is coherently consistent with the use of filtering as a strategy to enhance image properties, and to unveil otherwise undetectable information. Admittedly, this approach is not systematically adopted in radiomics, but it should be regarded as promising given that RFs derived from preprocessed CTs have been associated with oncological outcomes in cancers from other districts, such the oropharynx, a head and neck subsite whose incidence is rapidly increasing in highly-developed countries [23,24]. On this basis, and even regardless of the positive results, the use of image filtering can be considered among the strengths of this sub-study.

Additional pros include the high quality of the image dataset, which presents homogenous acquisition parameters and segmentations performed by a single observer. On the other hand, some limitations common to retrospective radiomic studies should be acknowledged, including the lack of external validation on the large dataset and the presence of missing data in the clinical dataset (e.g., histopathological assessment, smoking status). Specifically, this latter pitfall may have affected the results of the clinical models, leading to the exclusion of potential prognosticators. Another limitation is the fact that the majority of extracted features were obtained from small tumors (less than 2 cm) and are typically less stable; therefore, the possible influence of tumor size on the robustness of features remains to be investigated.

## 5. Conclusions

To conclude, the use of radiomics for outcome prediction in this clinical setting is promising, and results seem to be consistent across studies despite some methodological differences. The use of image filtering should be encouraged in the context of a well-defined pipeline, and will probably favor standardization efforts, such as a recent IBSI initiative [25]. Prospectively, further studies are being planned in our group to externally validate these findings in order to better determine the potential of RFs as non-invasive and reproducible biomarkers in ES-NSCLC [26].

## Figures and Tables

**Table 1:** Patient- and tumor-related clinical characteristics; all collected information is presented, including variable distribution for variables that were not included in outcome modeling. The sample size being equal to 100, percentages are not provided to avoid redundancy.

Characteristic	N = 100
**Median age at diagnosis, IQR**	76, 70–82
**Comorbidities**	
Yes	92
No	8
**CCI**	
CCI Median, IQR	7 (6, 8)
CCI range	2, 11
**Hypertension**	
Yes	61
No	39
**Heart disease**	
Yes	46
No	54
**Diabetes**	
Yes	19
No	81
**COPD**	
Yes	48
No	52
**Tobacco smoke**	
Never smoked	21
Active smoker	41
Former smoker	30
Unknown	8
**Baseline spirometry**	
Available	84
Not available	16
**Vital Capacity Median, IQR**	2.74, 2.00–3.28
Unknown	23
**FEV1 Median, IQR**	1.64, 1.21–2.18
Unknown	21
**PEF Median, IQR**	64.00, 46.00–83.00
Unknown	23
**DLCO Median, IQR**	55.00, 40.00–71.00
Unknown	25
**Vital Capacity % Median, IQR**	84.00, 71.00–93.00
Unknown	24
**FEV1% Median, IQR**	79.00, 55.00–92.00
Unknown	24
**Clinical Staging**	
IA1	12
IA2	46
IA3	16
IB	21
IIB	5
**Lesion side**	
Right	56
Left	44
**Central vs. Peripheral**	
Central	27
Peripheral	73
**Shape**	
Round	33
Oval	23
Complex	44
**Margins**	
Smooth	24
Lobulated	17
Spiculate/Irregular	59
**Peripheral GGO**	
Yes	42
No	58
**Density**	
Partially solid	30
Solid	70
**Internal air bronchogram**	
Yes	29
No	71
**Emphysema**	
Yes	35
No	65
**Periscissural location**	
Yes	23
No	77
**Pleuric contact**	
Yes	62
No	38

**Table 2:** Results of the multivariable analyses for clinical, radiomic, and clinico-radiomic models.

OVERALL SURVIVAL									
Characteristic	Clinical Model			Radiomic Model			Clinico-Radiomic Model		
N	HR	95% CI	*p*-value	HR	95% CI	*p*-value	HR	95% CI	*p*-value
BED_value									
<med 124.8 Gy	—	—					—	—	
>=med 124.8 Gy	0.08	0.01, 0.69	**0.022**				0.15	0.01, 3.11	0.221
radiomic_score_os				1.47	1.17, 1.86	**0.001**	1.46	1.12, 1.90	**0.006**
n = 96.0; N events = 6.00;									
**PROGRESSION-FREE SURVIVAL**									
**Characteristic**	**Clinical Model**			**Radiomic Model**			**Clinico-Radiomic Model**		
	HR	95% CI	*p*-value	HR	95% CI	*p*-value	HR	95% CI	*p*-value
FEV1%	0.98	0.97, 1.00	**0.020**				0.99	0.98, 1.00	0.181
Shape									
Round	—	—					—	—	
Complex	0.30	0.13, 0.71	**0.006**				0.40	0.17, 0.96	**0.039**
Oval	0.21	0.06, 0.75	**0.016**				0.27	0.07, 0.97	**0.046**
radiomic_score*10				1.45	1.25, 1.68	**<0.001**	1.35	1.13, 1.61	**<0.001**
n = 76.0; N events = 26.0;									
**LOCAL PROGRESSION-FREE SURVIVAL**									
**Characteristic**	**Clinical Model**			**Radiomic Model**			**Clinico-Radiomic Model**		
	HR	95% CI	*p*-value	HR	95% CI	*p*-value	HR	95% CI	*p*-value
FEV1%	0.97	0.95, 1.00	**0.019**				0.98	0.95, 1.00	0.096
radiomic_score_local				7.15	3.07, 16.7	<0.001	6.31	2.32, 17.1	**<0.001**
n = 76.0; N events = 12.0;									

Note: significant *p*-values are in bold.

**Table 3:** Summary of model performances (C-index Median and IQR) coming from repeated internal three-fold cross-validation for all considered outcomes and models.

OVERALL SURVIVAL					
Clinical Model		Radiomic Model		Clinico-Radiomic Model	
Train	Test	Train	Test	Train	Test
0.88 (0.85–0.91)	0.87 (0.86–0.88)	0.91 (0.90–0.92)	0.95 (0.93–0.97)	0.97 (0.95–0.98)	0.92 (0.91–0.93)
**PROGRESSION-FREE SURVIVAL**					
Clinical Model		Radiomic Model		Clinico-Radiomic Model	
Train	Test	Train	Test	Train	Test
0.70 (0.69–0.71)	0.68 (0.66–0.71)	0.68 (0.66–0.70)	0.67 (0.66–0.68)	0.76 (0.75–0.79)	0.73 (0.70–0.76)
**LOCAL PROGRESSION-FREE SURVIVAL**					
Clinical Model		Radiomic Model		Clinico-Radiomic Model	
Train	Test	Train	Test	Train	Test
0.79 (0.74–0.84)	0.71 (0.70–0.73)	0.78 (0.75–0.80)	0.77 (0.76–0.78)	0.86 (0.85–0.87)	0.84 (0.80–0.87)

## Data Availability

The raw data supporting the conclusions of this article will be made available by the authors on request.

## Supplementary Materials

The following supporting information can be downloaded at: https://www.mdpi.com/article/10.3390/cancers17050908/s1, Figure S1: Kaplan-Meier curves stratified according to the median BED value of the study population (namely, BED= 124.8 Gy, α/β= 10 Gy); Table S1: Overview and definition of the clinical variables selected for model inclusion due to their known clinical significance. Patient-, tumor- and treatment-related variables were included. Inclusion criteria also considered the number of missings (e.g., histopathological diagnosis, or the number of pack-year could not be included); Table S2: Fractionation schedules; Table S3: Patients’ follow-up: information of disease status at the last available follow-up, type of disease progression and type of treatment at disease progression; Table S4: List of all the extracted radiomic features.

## Abbreviations

4DCT	Four-Dimensional Computed Tomography
AJCC	American Joint Committee On Cancer
BED	Biologically Effective Dose
CCI	Charlson Comorbidity Index
CI	Confidence Interval
CT	Computed Tomography
DICOM	Digital Imaging And Communications In Medicine (DICOM)
ES-NSCLC	Early-Stage NSCLC
ES	Early Stage
FEV	Forced Expiratory Volume
GLCM	Grey Leve Co-Occurrence Matrix
GLDM	Grey Level Dependence Matrix
GLSZM	Grey Level Size Zone Matrix
H	High
HR	Hazard Ratio
IQR	Interquartile Range
L	Low
LASSO	Least Absolute Shrinkage And Selection Operator
LRPFS	Local Progression Free-Survival
LRPFS	Loco-Regional Progression-Free Survival
NSCLC	Non-Small Cell Lung Cancer
OS	Overall Survival
OTT	Overall Treatment Time
PFS	Progression-Free Survival
PH	Proportional Hazard
RF	Radiomic Features
RT	Radiation Therapy
SBRT	Stereotactic Body Radiotherapy
TNM	Tumor, Node, Metastasis
