# Supplementary material for: Harnessing Baseline Radiomic Features in Early-Stage NSCLC: What Role in Clinical Outcome Modeling for SBRT Candidates?
Source: Cancers (Basel). 2025 Mar 6;17(5):908. doi: 10.3390/cancers17050908 (PMC11899142; doi:10.3390/cancers17050908)
Supplement: Supplementary file 1 [file cancers-17-00908-s001.zip › cancers-3443067-supplementary.pdf]

Supplementary materials

Table S1. Overview and definition of the clinical variables selected for model inclusion due to their known clinical significance. Patient-, tumor- and treatment-related variables were included. Inclusion criteria also considered the number of missings (e.g. histopathological diagnosis, or the number of pack-year could not be included).

Variable	Definition
**Age at diagnosis**	The age at which diagnosis of ES-NSCLC was first made; the date of histopathological assessment has been used, whenever available
**Comorbidities**	The presence of any concomitant, clinically-relevant, non-oncological disease (e.g. heart failure, renal failure, COPD)
**Charlson Comorbidity Index (CCI)**	It is a validated score used to categorize patients' comorbidities per the standardized International Classification of Diseases (ICD) ontology. Each of the 17 comorbity category is assigned with a weight from 1 to 6, based on the adjusted risk of mortality
**Heart disease**	The presence of any heart pathological condition requiring medication (e.g. antihypertensives, diuretics)
**Diabetes mellitus**	The presence of medically-established diagnosis of diabetes, requiring either a specific diet or medication
**Tobacco smoke**	The former or current presence of tobacco habit
**Lesion shape**	The simplified shape of the primary tumor, classified as either round, oval, or complex
**Lesion Margins**	The simplified margins of the primary tumor, classified as either smooth, lobulated or irregular
**DLCO**	A spirometry parameter measuring the diffusing capacity of the lung parenchyma for carbon monoxyde; values ≥ 75 cc of CO/sec/mm are considered as normal
**Forced Expiratory Volume in 1 second, % (FEV1%)**	A spirometry parameter measuring the amount of air that a person can force out of their lungs in 1 second; values ≥ 80% are considered as normal
**Clinical staging**	The tumor staging according to the AJCC TNM 8th classification
**BED**	The biological effective dose, in Gy, considering an α/β ratio of 10 Gy
**OTT**	Overall treatment time, which is the time between treatment start and treatment completion, considering inter-fraction intervals, if any

Table S2. Fractionation schedules

Dose/fraction (Gy)	Number of fractions	Total dose	BED (Gy)	Number of patients
7	5	35	59,5	2
8	5	40	72,0	1
10	5	50	100,0	9
11	5	55	115,5	2
13	3	39	89,7	1
15	3	45	112,5	36
16	3	48	124,8	2
18	3	54	151,2	29
20	3	60	180,0	18

Table S3. Patients' follow-up: information of disease status at the last available follow-up, type of disease progression and type of treatment at disease progression.

Variable	n (%)
**Status at the last follow-up (n= 100)**	
Non-evidence of disease	76
Alive with disease	17
Dead (any cause)	7
**Type of progression (n= 31)**	
In-field	9 (29)
Out-field	22 (71)
**PD out-field location (n= 22)**	
Ipsilateral lung	5 (23)
Contralateral lung	5 (23)
Mediastinum	9 (41)
Bone	1 (5)
Visceral	2 (9)
**Treatment at PD (n= 31)**	
Surgery	1 (3)
SBRT (non-irradiation)	13 (42)
Re-irradiation	1 (3)
Chemo-RT combinations (induction or concomitant)	2 (6)
Systemic treatment only	3 (10)
Follow-up	2 (6)
Best supportive care	2 (6)
Unknown	7 (23)

Figure S1. ***Kaplan-Meier curves stratified according to the median BED value of the study population (namely, BED= 124.8 Gy, α/β= 10 Gy).***

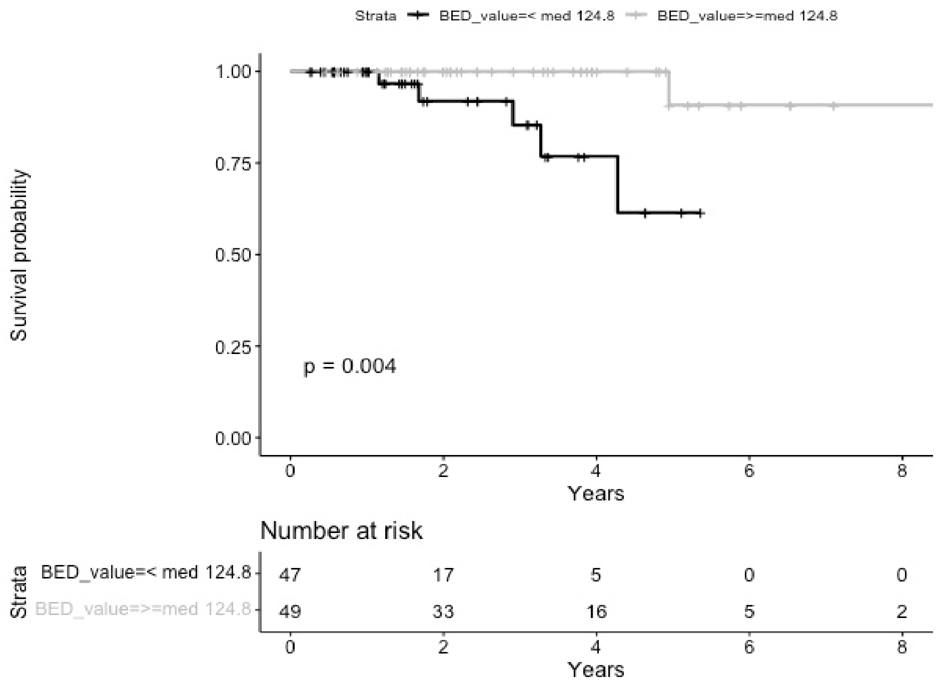

Table S4. List of all the extracted radiomic features

original_shape_Elongation
original_shape_Flatness
original_shape_LeastAxisLength
original_shape_MajorAxisLength
original_shape_Maximum2DDiameterColumn
original_shape_Maximum2DDiameterRow
original_shape_Maximum2DDiameterSlice
original_shape_Maximum3DDiameter
original_shape_MeshVolume
original_shape_MinorAxisLength
original_shape_Sphericity
original_shape_SurfaceArea
original_shape_SurfaceVolumeRatio
original_shape_VoxelVolume
original_firstorder_10Percentile
original_firstorder_90Percentile
original_firstorder_Energy
original_firstorder_Entropy
original_firstorder_InterquartileRange
original_firstorder_Kurtosis
original_firstorder_Maximum
original_firstorder_MeanAbsoluteDeviation
original_firstorder_Mean
original_firstorder_Median
original_firstorder_Minimum
original_firstorder_Range
original_firstorder_RobustMeanAbsoluteDeviation
original_firstorder_RootMeanSquared
original_firstorder_Skewness
original_firstorder_TotalEnergy
original_firstorder_Uniformity
original_firstorder_Variance
original_glcm_Autocorrelation
original_glcm_ClusterProminence
original_glcm_ClusterShade
original_glcm_ClusterTendency
original_glcm_Contrast
original_glcm_Correlation
original_glcm_DifferenceAverage
original_glcm_DifferenceEntropy
original_glcm_DifferenceVariance
original_glcm_Id
original_glcm_Idm
original_glcm_Idmn
original_glcm_Idn

original_glcm_Imc1
original_glcm_Imc2
original_glcm_InverseVariance
original_glcm_JointAverage
original_glcm_JointEnergy
original_glcm_JointEntropy
original_glcm_MCC
original_glcm_MaximumProbability
original_glcm_SumAverage
original_glcm_SumEntropy
original_glcm_SumSquares
original_gldm_DependenceEntropy
original_gldm_DependenceNonUniformity
original_gldm_DependenceNonUniformityNormalized
original_gldm_DependenceVariance
original_gldm_GrayLevelNonUniformity
original_gldm_GrayLevelVariance
original_gldm_HighGrayLevelEmphasis
original_gldm_LargeDependenceEmphasis
original_gldm_LargeDependenceHighGrayLevelEmphasis
original_gldm_LargeDependenceLowGrayLevelEmphasis
original_gldm_LowGrayLevelEmphasis
original_gldm_SmallDependenceEmphasis
original_gldm_SmallDependenceHighGrayLevelEmphasis
original_gldm_SmallDependenceLowGrayLevelEmphasis
original_glrlm_GrayLevelNonUniformity
original_glrlm_GrayLevelNonUniformityNormalized
original_glrlm_GrayLevelVariance
original_glrlm_HighGrayLevelRunEmphasis
original_glrlm_LongRunEmphasis
original_glrlm_LongRunHighGrayLevelEmphasis
original_glrlm_LongRunLowGrayLevelEmphasis
original_glrlm_LowGrayLevelRunEmphasis
original_glrlm_RunEntropy
original_glrlm_RunLengthNonUniformity
original_glrlm_RunLengthNonUniformityNormalized
original_glrlm_RunPercentage
original_glrlm_RunVariance
original_glrlm_ShortRunEmphasis
original_glrlm_ShortRunHighGrayLevelEmphasis
original_glrlm_ShortRunLowGrayLevelEmphasis
original_glszm_GrayLevelNonUniformity
original_glszm_GrayLevelNonUniformityNormalized
original_glszm_GrayLevelVariance
original_glszm_HighGrayLevelZoneEmphasis
original_glszm_LargeAreaEmphasis
original_glszm_LargeAreaHighGrayLevelEmphasis

original_glszm_LargeAreaLowGrayLevelEmphasis
original_glszm_LowGrayLevelZoneEmphasis
original_glszm_SizeZoneNonUniformity
original_glszm_SizeZoneNonUniformityNormalized
original_glszm_SmallAreaEmphasis
original_glszm_SmallAreaHighGrayLevelEmphasis
original_glszm_SmallAreaLowGrayLevelEmphasis
original_glszm_ZoneEntropy
original_glszm_ZonePercentage
original_glszm_ZoneVariance
original_ngtdm_Busyness
original_ngtdm_Coarseness
original_ngtdm_Complexity
original_ngtdm_Contrast
original_ngtdm_Strength
exponential_firstorder_10Percentile
exponential_firstorder_90Percentile
exponential_firstorder_Energy
exponential_firstorder_Entropy
exponential_firstorder_InterquartileRange
exponential_firstorder_Kurtosis
exponential_firstorder_Maximum
exponential_firstorder_MeanAbsoluteDeviation
exponential_firstorder_Mean
exponential_firstorder_Median
exponential_firstorder_Minimum
exponential_firstorder_Range
exponential_firstorder_RobustMeanAbsoluteDeviation
exponential_firstorder_RootMeanSquared
exponential_firstorder_Skewness
exponential_firstorder_TotalEnergy
exponential_firstorder_Uniformity
exponential_firstorder_Variance
exponential_glcm_Autocorrelation
exponential_glcm_ClusterProminence
exponential_glcm_ClusterShade
exponential_glcm_ClusterTendency
exponential_glcm_Contrast
exponential_glcm_Correlation
exponential_glcm_DifferenceAverage
exponential_glcm_DifferenceEntropy
exponential_glcm_DifferenceVariance
exponential_glcm_Id
exponential_glcm_Idm
exponential_glcm_Idmn
exponential_glcm_Idn
exponential_glcm_Imc1

exponential_glcm_Imc2
exponential_glcm_InverseVariance
exponential_glcm_JointAverage
exponential_glcm_JointEnergy
exponential_glcm_JointEntropy
exponential_glcm_MCC
exponential_glcm_MaximumProbability
exponential_glcm_SumAverage
exponential_glcm_SumEntropy
exponential_glcm_SumSquares
exponential_gldm_DependenceEntropy
exponential_gldm_DependenceNonUniformity
exponential_gldm_DependenceNonUniformityNormalized
exponential_gldm_DependenceVariance
exponential_gldm_GrayLevelNonUniformity
exponential_gldm_GrayLevelVariance
exponential_gldm_HighGrayLevelEmphasis
exponential_gldm_LargeDependenceEmphasis
exponential_gldm_LargeDependenceHighGrayLevelEmphasis
exponential_gldm_LargeDependenceLowGrayLevelEmphasis
exponential_gldm_LowGrayLevelEmphasis
exponential_gldm_SmallDependenceEmphasis
exponential_gldm_SmallDependenceHighGrayLevelEmphasis
exponential_gldm_SmallDependenceLowGrayLevelEmphasis
exponential_glrlm_GrayLevelNonUniformity
exponential_glrlm_GrayLevelNonUniformityNormalized
exponential_glrlm_GrayLevelVariance
exponential_glrlm_HighGrayLevelRunEmphasis
exponential_glrlm_LongRunEmphasis
exponential_glrlm_LongRunHighGrayLevelEmphasis
exponential_glrlm_LongRunLowGrayLevelEmphasis
exponential_glrlm_LowGrayLevelRunEmphasis
exponential_glrlm_RunEntropy
exponential_glrlm_RunLengthNonUniformity
exponential_glrlm_RunLengthNonUniformityNormalized
exponential_glrlm_RunPercentage
exponential_glrlm_RunVariance
exponential_glrlm_ShortRunEmphasis
exponential_glrlm_ShortRunHighGrayLevelEmphasis
exponential_glrlm_ShortRunLowGrayLevelEmphasis
exponential_glszm_GrayLevelNonUniformity
exponential_glszm_GrayLevelNonUniformityNormalized
exponential_glszm_GrayLevelVariance
exponential_glszm_HighGrayLevelZoneEmphasis
exponential_glszm_LargeAreaEmphasis
exponential_glszm_LargeAreaHighGrayLevelEmphasis
exponential_glszm_LargeAreaLowGrayLevelEmphasis

exponential_glszm_LowGrayLevelZoneEmphasis
exponential_glszm_SizeZoneNonUniformity
exponential_glszm_SizeZoneNonUniformityNormalized
exponential_glszm_SmallAreaEmphasis
exponential_glszm_SmallAreaHighGrayLevelEmphasis
exponential_glszm_SmallAreaLowGrayLevelEmphasis
exponential_glszm_ZoneEntropy
exponential_glszm_ZonePercentage
exponential_glszm_ZoneVariance
exponential_ngtdm_Busyness
exponential_ngtdm_Coarseness
exponential_ngtdm_Complexity
exponential_ngtdm_Contrast
exponential_ngtdm_Strength
gradient_firstorder_10Percentile
gradient_firstorder_90Percentile
gradient_firstorder_Energy
gradient_firstorder_Entropy
gradient_firstorder_InterquartileRange
gradient_firstorder_Kurtosis
gradient_firstorder_Maximum
gradient_firstorder_MeanAbsoluteDeviation
gradient_firstorder_Mean
gradient_firstorder_Median
gradient_firstorder_Minimum
gradient_firstorder_Range
gradient_firstorder_RobustMeanAbsoluteDeviation
gradient_firstorder_RootMeanSquared
gradient_firstorder_Skewness
gradient_firstorder_TotalEnergy
gradient_firstorder_Uniformity
gradient_firstorder_Variance
gradient_glcm_Autocorrelation
gradient_glcm_ClusterProminence
gradient_glcm_ClusterShade
gradient_glcm_ClusterTendency
gradient_glcm_Contrast
gradient_glcm_Correlation
gradient_glcm_DifferenceAverage
gradient_glcm_DifferenceEntropy
gradient_glcm_DifferenceVariance
gradient_glcm_Id
gradient_glcm_Idm
gradient_glcm_Idmn
gradient_glcm_Idn
gradient_glcm_Imc1
gradient_glcm_Imc2

gradient_glcm_InverseVariance
gradient_glcm_JointAverage
gradient_glcm_JointEnergy
gradient_glcm_JointEntropy
gradient_glcm_MCC
gradient_glcm_MaximumProbability
gradient_glcm_SumAverage
gradient_glcm_SumEntropy
gradient_glcm_SumSquares
gradient_gldm_DependenceEntropy
gradient_gldm_DependenceNonUniformity
gradient_gldm_DependenceNonUniformityNormalized
gradient_gldm_DependenceVariance
gradient_gldm_GrayLevelNonUniformity
gradient_gldm_GrayLevelVariance
gradient_gldm_HighGrayLevelEmphasis
gradient_gldm_LargeDependenceEmphasis
gradient_gldm_LargeDependenceHighGrayLevelEmphasis
gradient_gldm_LargeDependenceLowGrayLevelEmphasis
gradient_gldm_LowGrayLevelEmphasis
gradient_gldm_SmallDependenceEmphasis
gradient_gldm_SmallDependenceHighGrayLevelEmphasis
gradient_gldm_SmallDependenceLowGrayLevelEmphasis
gradient_glrlm_GrayLevelNonUniformity
gradient_glrlm_GrayLevelNonUniformityNormalized
gradient_glrlm_GrayLevelVariance
gradient_glrlm_HighGrayLevelRunEmphasis
gradient_glrlm_LongRunEmphasis
gradient_glrlm_LongRunHighGrayLevelEmphasis
gradient_glrlm_LongRunLowGrayLevelEmphasis
gradient_glrlm_LowGrayLevelRunEmphasis
gradient_glrlm_RunEntropy
gradient_glrlm_RunLengthNonUniformity
gradient_glrlm_RunLengthNonUniformityNormalized
gradient_glrlm_RunPercentage
gradient_glrlm_RunVariance
gradient_glrlm_ShortRunEmphasis
gradient_glrlm_ShortRunHighGrayLevelEmphasis
gradient_glrlm_ShortRunLowGrayLevelEmphasis
gradient_glszm_GrayLevelNonUniformity
gradient_glszm_GrayLevelNonUniformityNormalized
gradient_glszm_GrayLevelVariance
gradient_glszm_HighGrayLevelZoneEmphasis
gradient_glszm_LargeAreaEmphasis
gradient_glszm_LargeAreaHighGrayLevelEmphasis
gradient_glszm_LargeAreaLowGrayLevelEmphasis
gradient_glszm_LowGrayLevelZoneEmphasis

gradient_glszm_SizeZoneNonUniformity
gradient_glszm_SizeZoneNonUniformityNormalized
gradient_glszm_SmallAreaEmphasis
gradient_glszm_SmallAreaHighGrayLevelEmphasis
gradient_glszm_SmallAreaLowGrayLevelEmphasis
gradient_glszm_ZoneEntropy
gradient_glszm_ZonePercentage
gradient_glszm_ZoneVariance
gradient_ngtdm_Busyness
gradient_ngtdm_Coarseness
gradient_ngtdm_Complexity
gradient_ngtdm_Contrast
gradient_ngtdm_Strength
lbp-2D_firstorder_10Percentile
lbp-2D_firstorder_90Percentile
lbp-2D_firstorder_Energy
lbp-2D_firstorder_Entropy
lbp-2D_firstorder_InterquartileRange
lbp-2D_firstorder_Kurtosis
lbp-2D_firstorder_Maximum
lbp-2D_firstorder_MeanAbsoluteDeviation
lbp-2D_firstorder_Mean
lbp-2D_firstorder_Median
lbp-2D_firstorder_Minimum
lbp-2D_firstorder_Range
lbp-2D_firstorder_RobustMeanAbsoluteDeviation
lbp-2D_firstorder_RootMeanSquared
lbp-2D_firstorder_Skewness
lbp-2D_firstorder_TotalEnergy
lbp-2D_firstorder_Uniformity
lbp-2D_firstorder_Variance
lbp-2D_glcm_Autocorrelation
lbp-2D_glcm_ClusterProminence
lbp-2D_glcm_ClusterShade
lbp-2D_glcm_ClusterTendency
lbp-2D_glcm_Contrast
lbp-2D_glcm_Correlation
lbp-2D_glcm_DifferenceAverage
lbp-2D_glcm_DifferenceEntropy
lbp-2D_glcm_DifferenceVariance
lbp-2D_glcm_Id
lbp-2D_glcm_Idm
lbp-2D_glcm_Idmn
lbp-2D_glcm_Idn
lbp-2D_glcm_Imc1
lbp-2D_glcm_Imc2
lbp-2D_glcm_InverseVariance

lbp-2D_glcm_JointAverage
lbp-2D_glcm_JointEnergy
lbp-2D_glcm_JointEntropy
lbp-2D_glcm_MCC
lbp-2D_glcm_MaximumProbability
lbp-2D_glcm_SumAverage
lbp-2D_glcm_SumEntropy
lbp-2D_glcm_SumSquares
lbp-2D_gldm_DependenceEntropy
lbp-2D_gldm_DependenceNonUniformity
lbp-2D_gldm_DependenceNonUniformityNormalized
lbp-2D_gldm_DependenceVariance
lbp-2D_gldm_GrayLevelNonUniformity
lbp-2D_gldm_GrayLevelVariance
lbp-2D_gldm_HighGrayLevelEmphasis
lbp-2D_gldm_LargeDependenceEmphasis
lbp-2D_gldm_LargeDependenceHighGrayLevelEmphasis
lbp-2D_gldm_LargeDependenceLowGrayLevelEmphasis
lbp-2D_gldm_LowGrayLevelEmphasis
lbp-2D_gldm_SmallDependenceEmphasis
lbp-2D_gldm_SmallDependenceHighGrayLevelEmphasis
lbp-2D_gldm_SmallDependenceLowGrayLevelEmphasis
lbp-2D_glrlm_GrayLevelNonUniformity
lbp-2D_glrlm_GrayLevelNonUniformityNormalized
lbp-2D_glrlm_GrayLevelVariance
lbp-2D_glrlm_HighGrayLevelRunEmphasis
lbp-2D_glrlm_LongRunEmphasis
lbp-2D_glrlm_LongRunHighGrayLevelEmphasis
lbp-2D_glrlm_LongRunLowGrayLevelEmphasis
lbp-2D_glrlm_LowGrayLevelRunEmphasis
lbp-2D_glrlm_RunEntropy
lbp-2D_glrlm_RunLengthNonUniformity
lbp-2D_glrlm_RunLengthNonUniformityNormalized
lbp-2D_glrlm_RunPercentage
lbp-2D_glrlm_RunVariance
lbp-2D_glrlm_ShortRunEmphasis
lbp-2D_glrlm_ShortRunHighGrayLevelEmphasis
lbp-2D_glrlm_ShortRunLowGrayLevelEmphasis
lbp-2D_glszm_GrayLevelNonUniformity
lbp-2D_glszm_GrayLevelNonUniformityNormalized
lbp-2D_glszm_GrayLevelVariance
lbp-2D_glszm_HighGrayLevelZoneEmphasis
lbp-2D_glszm_LargeAreaEmphasis
lbp-2D_glszm_LargeAreaHighGrayLevelEmphasis
lbp-2D_glszm_LargeAreaLowGrayLevelEmphasis
lbp-2D_glszm_LowGrayLevelZoneEmphasis
lbp-2D_glszm_SizeZoneNonUniformity

lbp-2D_glszm_SizeZoneNonUniformityNormalized
lbp-2D_glszm_SmallAreaEmphasis
lbp-2D_glszm_SmallAreaHighGrayLevelEmphasis
lbp-2D_glszm_SmallAreaLowGrayLevelEmphasis
lbp-2D_glszm_ZoneEntropy
lbp-2D_glszm_ZonePercentage
lbp-2D_glszm_ZoneVariance
lbp-2D_ngtdm_Busyness
lbp-2D_ngtdm_Coarseness
lbp-2D_ngtdm_Complexity
lbp-2D_ngtdm_Contrast
lbp-2D_ngtdm_Strength
lbp-3D-m1_firstorder_10Percentile
lbp-3D-m1_firstorder_90Percentile
lbp-3D-m1_firstorder_Energy
lbp-3D-m1_firstorder_Entropy
lbp-3D-m1_firstorder_InterquartileRange
lbp-3D-m1_firstorder_Kurtosis
lbp-3D-m1_firstorder_Maximum
lbp-3D-m1_firstorder_MeanAbsoluteDeviation
lbp-3D-m1_firstorder_Mean
lbp-3D-m1_firstorder_Median
lbp-3D-m1_firstorder_Minimum
lbp-3D-m1_firstorder_Range
lbp-3D-m1_firstorder_RobustMeanAbsoluteDeviation
lbp-3D-m1_firstorder_RootMeanSquared
lbp-3D-m1_firstorder_Skewness
lbp-3D-m1_firstorder_TotalEnergy
lbp-3D-m1_firstorder_Uniformity
lbp-3D-m1_firstorder_Variance
lbp-3D-m1_glcm_Autocorrelation
lbp-3D-m1_glcm_ClusterProminence
lbp-3D-m1_glcm_ClusterShade
lbp-3D-m1_glcm_ClusterTendency
lbp-3D-m1_glcm_Contrast
lbp-3D-m1_glcm_Correlation
lbp-3D-m1_glcm_DifferenceAverage
lbp-3D-m1_glcm_DifferenceEntropy
lbp-3D-m1_glcm_DifferenceVariance
lbp-3D-m1_glcm_Id
lbp-3D-m1_glcm_Idm
lbp-3D-m1_glcm_Idmn
lbp-3D-m1_glcm_Idn
lbp-3D-m1_glcm_Imc1
lbp-3D-m1_glcm_Imc2
lbp-3D-m1_glcm_InverseVariance
lbp-3D-m1_glcm_JointAverage

lbp-3D-m1_glcm_JointEnergy
lbp-3D-m1_glcm_JointEntropy
lbp-3D-m1_glcm_MCC
lbp-3D-m1_glcm_MaximumProbability
lbp-3D-m1_glcm_SumAverage
lbp-3D-m1_glcm_SumEntropy
lbp-3D-m1_glcm_SumSquares
lbp-3D-m1_gldm_DependenceEntropy
lbp-3D-m1_gldm_DependenceNonUniformity
lbp-3D-m1_gldm_DependenceNonUniformityNormalized
lbp-3D-m1_gldm_DependenceVariance
lbp-3D-m1_gldm_GrayLevelNonUniformity
lbp-3D-m1_gldm_GrayLevelVariance
lbp-3D-m1_gldm_HighGrayLevelEmphasis
lbp-3D-m1_gldm_LargeDependenceEmphasis
lbp-3D-m1_gldm_LargeDependenceHighGrayLevelEmphasis
lbp-3D-m1_gldm_LargeDependenceLowGrayLevelEmphasis
lbp-3D-m1_gldm_LowGrayLevelEmphasis
lbp-3D-m1_gldm_SmallDependenceEmphasis
lbp-3D-m1_gldm_SmallDependenceHighGrayLevelEmphasis
lbp-3D-m1_gldm_SmallDependenceLowGrayLevelEmphasis
lbp-3D-m1_glrlm_GrayLevelNonUniformity
lbp-3D-m1_glrlm_GrayLevelNonUniformityNormalized
lbp-3D-m1_glrlm_GrayLevelVariance
lbp-3D-m1_glrlm_HighGrayLevelRunEmphasis
lbp-3D-m1_glrlm_LongRunEmphasis
lbp-3D-m1_glrlm_LongRunHighGrayLevelEmphasis
lbp-3D-m1_glrlm_LongRunLowGrayLevelEmphasis
lbp-3D-m1_glrlm_LowGrayLevelRunEmphasis
lbp-3D-m1_glrlm_RunEntropy
lbp-3D-m1_glrlm_RunLengthNonUniformity
lbp-3D-m1_glrlm_RunLengthNonUniformityNormalized
lbp-3D-m1_glrlm_RunPercentage
lbp-3D-m1_glrlm_RunVariance
lbp-3D-m1_glrlm_ShortRunEmphasis
lbp-3D-m1_glrlm_ShortRunHighGrayLevelEmphasis
lbp-3D-m1_glrlm_ShortRunLowGrayLevelEmphasis
lbp-3D-m1_glszm_GrayLevelNonUniformity
lbp-3D-m1_glszm_GrayLevelNonUniformityNormalized
lbp-3D-m1_glszm_GrayLevelVariance
lbp-3D-m1_glszm_HighGrayLevelZoneEmphasis
lbp-3D-m1_glszm_LargeAreaEmphasis
lbp-3D-m1_glszm_LargeAreaHighGrayLevelEmphasis
lbp-3D-m1_glszm_LargeAreaLowGrayLevelEmphasis
lbp-3D-m1_glszm_LowGrayLevelZoneEmphasis
lbp-3D-m1_glszm_SizeZoneNonUniformity
lbp-3D-m1_glszm_SizeZoneNonUniformityNormalized

lbp-3D-m1_glszm_SmallAreaEmphasis
lbp-3D-m1_glszm_SmallAreaHighGrayLevelEmphasis
lbp-3D-m1_glszm_SmallAreaLowGrayLevelEmphasis
lbp-3D-m1_glszm_ZoneEntropy
lbp-3D-m1_glszm_ZonePercentage
lbp-3D-m1_glszm_ZoneVariance
lbp-3D-m1_ngtdm_Busyness
lbp-3D-m1_ngtdm_Coarseness
lbp-3D-m1_ngtdm_Complexity
lbp-3D-m1_ngtdm_Contrast
lbp-3D-m1_ngtdm_Strength
lbp-3D-m2_firstorder_10Percentile
lbp-3D-m2_firstorder_90Percentile
lbp-3D-m2_firstorder_Energy
lbp-3D-m2_firstorder_Entropy
lbp-3D-m2_firstorder_InterquartileRange
lbp-3D-m2_firstorder_Kurtosis
lbp-3D-m2_firstorder_Maximum
lbp-3D-m2_firstorder_MeanAbsoluteDeviation
lbp-3D-m2_firstorder_Mean
lbp-3D-m2_firstorder_Median
lbp-3D-m2_firstorder_Minimum
lbp-3D-m2_firstorder_Range
lbp-3D-m2_firstorder_RobustMeanAbsoluteDeviation
lbp-3D-m2_firstorder_RootMeanSquared
lbp-3D-m2_firstorder_Skewness
lbp-3D-m2_firstorder_TotalEnergy
lbp-3D-m2_firstorder_Uniformity
lbp-3D-m2_firstorder_Variance
lbp-3D-m2_glcm_Autocorrelation
lbp-3D-m2_glcm_ClusterProminence
lbp-3D-m2_glcm_ClusterShade
lbp-3D-m2_glcm_ClusterTendency
lbp-3D-m2_glcm_Contrast
lbp-3D-m2_glcm_Correlation
lbp-3D-m2_glcm_DifferenceAverage
lbp-3D-m2_glcm_DifferenceEntropy
lbp-3D-m2_glcm_DifferenceVariance
lbp-3D-m2_glcm_Id
lbp-3D-m2_glcm_Idm
lbp-3D-m2_glcm_Idmn
lbp-3D-m2_glcm_Idn
lbp-3D-m2_glcm_Imc1
lbp-3D-m2_glcm_Imc2
lbp-3D-m2_glcm_InverseVariance
lbp-3D-m2_glcm_JointAverage
lbp-3D-m2_glcm_JointEnergy

lbp-3D-m2_glcm_JointEntropy
lbp-3D-m2_glcm_MCC
lbp-3D-m2_glcm_MaximumProbability
lbp-3D-m2_glcm_SumAverage
lbp-3D-m2_glcm_SumEntropy
lbp-3D-m2_glcm_SumSquares
lbp-3D-m2_gldm_DependenceEntropy
lbp-3D-m2_gldm_DependenceNonUniformity
lbp-3D-m2_gldm_DependenceNonUniformityNormalized
lbp-3D-m2_gldm_DependenceVariance
lbp-3D-m2_gldm_GrayLevelNonUniformity
lbp-3D-m2_gldm_GrayLevelVariance
lbp-3D-m2_gldm_HighGrayLevelEmphasis
lbp-3D-m2_gldm_LargeDependenceEmphasis
lbp-3D-m2_gldm_LargeDependenceHighGrayLevelEmphasis
lbp-3D-m2_gldm_LargeDependenceLowGrayLevelEmphasis
lbp-3D-m2_gldm_LowGrayLevelEmphasis
lbp-3D-m2_gldm_SmallDependenceEmphasis
lbp-3D-m2_gldm_SmallDependenceHighGrayLevelEmphasis
lbp-3D-m2_gldm_SmallDependenceLowGrayLevelEmphasis
lbp-3D-m2_glrlm_GrayLevelNonUniformity
lbp-3D-m2_glrlm_GrayLevelNonUniformityNormalized
lbp-3D-m2_glrlm_GrayLevelVariance
lbp-3D-m2_glrlm_HighGrayLevelRunEmphasis
lbp-3D-m2_glrlm_LongRunEmphasis
lbp-3D-m2_glrlm_LongRunHighGrayLevelEmphasis
lbp-3D-m2_glrlm_LongRunLowGrayLevelEmphasis
lbp-3D-m2_glrlm_LowGrayLevelRunEmphasis
lbp-3D-m2_glrlm_RunEntropy
lbp-3D-m2_glrlm_RunLengthNonUniformity
lbp-3D-m2_glrlm_RunLengthNonUniformityNormalized
lbp-3D-m2_glrlm_RunPercentage
lbp-3D-m2_glrlm_RunVariance
lbp-3D-m2_glrlm_ShortRunEmphasis
lbp-3D-m2_glrlm_ShortRunHighGrayLevelEmphasis
lbp-3D-m2_glrlm_ShortRunLowGrayLevelEmphasis
lbp-3D-m2_glszm_GrayLevelNonUniformity
lbp-3D-m2_glszm_GrayLevelNonUniformityNormalized
lbp-3D-m2_glszm_GrayLevelVariance
lbp-3D-m2_glszm_HighGrayLevelZoneEmphasis
lbp-3D-m2_glszm_LargeAreaEmphasis
lbp-3D-m2_glszm_LargeAreaHighGrayLevelEmphasis
lbp-3D-m2_glszm_LargeAreaLowGrayLevelEmphasis
lbp-3D-m2_glszm_LowGrayLevelZoneEmphasis
lbp-3D-m2_glszm_SizeZoneNonUniformity
lbp-3D-m2_glszm_SizeZoneNonUniformityNormalized
lbp-3D-m2_glszm_SmallAreaEmphasis

lbp-3D-m2_glszm_SmallAreaHighGrayLevelEmphasis
lbp-3D-m2_glszm_SmallAreaLowGrayLevelEmphasis
lbp-3D-m2_glszm_ZoneEntropy
lbp-3D-m2_glszm_ZonePercentage
lbp-3D-m2_glszm_ZoneVariance
lbp-3D-m2_ngtdm_Busyness
lbp-3D-m2_ngtdm_Coarseness
lbp-3D-m2_ngtdm_Complexity
lbp-3D-m2_ngtdm_Contrast
lbp-3D-m2_ngtdm_Strength
lbp-3D-k_firstorder_10Percentile
lbp-3D-k_firstorder_90Percentile
lbp-3D-k_firstorder_Energy
lbp-3D-k_firstorder_Entropy
lbp-3D-k_firstorder_InterquartileRange
lbp-3D-k_firstorder_Kurtosis
lbp-3D-k_firstorder_Maximum
lbp-3D-k_firstorder_MeanAbsoluteDeviation
lbp-3D-k_firstorder_Mean
lbp-3D-k_firstorder_Median
lbp-3D-k_firstorder_Minimum
lbp-3D-k_firstorder_Range
lbp-3D-k_firstorder_RobustMeanAbsoluteDeviation
lbp-3D-k_firstorder_RootMeanSquared
lbp-3D-k_firstorder_Skewness
lbp-3D-k_firstorder_TotalEnergy
lbp-3D-k_firstorder_Uniformity
lbp-3D-k_firstorder_Variance
lbp-3D-k_glcm_Autocorrelation
lbp-3D-k_glcm_ClusterProminence
lbp-3D-k_glcm_ClusterShade
lbp-3D-k_glcm_ClusterTendency
lbp-3D-k_glcm_Contrast
lbp-3D-k_glcm_Correlation
lbp-3D-k_glcm_DifferenceAverage
lbp-3D-k_glcm_DifferenceEntropy
lbp-3D-k_glcm_DifferenceVariance
lbp-3D-k_glcm_Id
lbp-3D-k_glcm_Idm
lbp-3D-k_glcm_Idmn
lbp-3D-k_glcm_Idn
lbp-3D-k_glcm_Imc1
lbp-3D-k_glcm_Imc2
lbp-3D-k_glcm_InverseVariance
lbp-3D-k_glcm_JointAverage
lbp-3D-k_glcm_JointEnergy
lbp-3D-k_glcm_JointEntropy

lbp-3D-k_glcm_MCC
lbp-3D-k_glcm_MaximumProbability
lbp-3D-k_glcm_SumAverage
lbp-3D-k_glcm_SumEntropy
lbp-3D-k_glcm_SumSquares
lbp-3D-k_gldm_DependenceEntropy
lbp-3D-k_gldm_DependenceNonUniformity
lbp-3D-k_gldm_DependenceNonUniformityNormalized
lbp-3D-k_gldm_DependenceVariance
lbp-3D-k_gldm_GrayLevelNonUniformity
lbp-3D-k_gldm_GrayLevelVariance
lbp-3D-k_gldm_HighGrayLevelEmphasis
lbp-3D-k_gldm_LargeDependenceEmphasis
lbp-3D-k_gldm_LargeDependenceHighGrayLevelEmphasis
lbp-3D-k_gldm_LargeDependenceLowGrayLevelEmphasis
lbp-3D-k_gldm_LowGrayLevelEmphasis
lbp-3D-k_gldm_SmallDependenceEmphasis
lbp-3D-k_gldm_SmallDependenceHighGrayLevelEmphasis
lbp-3D-k_gldm_SmallDependenceLowGrayLevelEmphasis
lbp-3D-k_glrlm_GrayLevelNonUniformity
lbp-3D-k_glrlm_GrayLevelNonUniformityNormalized
lbp-3D-k_glrlm_GrayLevelVariance
lbp-3D-k_glrlm_HighGrayLevelRunEmphasis
lbp-3D-k_glrlm_LongRunEmphasis
lbp-3D-k_glrlm_LongRunHighGrayLevelEmphasis
lbp-3D-k_glrlm_LongRunLowGrayLevelEmphasis
lbp-3D-k_glrlm_LowGrayLevelRunEmphasis
lbp-3D-k_glrlm_RunEntropy
lbp-3D-k_glrlm_RunLengthNonUniformity
lbp-3D-k_glrlm_RunLengthNonUniformityNormalized
lbp-3D-k_glrlm_RunPercentage
lbp-3D-k_glrlm_RunVariance
lbp-3D-k_glrlm_ShortRunEmphasis
lbp-3D-k_glrlm_ShortRunHighGrayLevelEmphasis
lbp-3D-k_glrlm_ShortRunLowGrayLevelEmphasis
lbp-3D-k_glszm_GrayLevelNonUniformity
lbp-3D-k_glszm_GrayLevelNonUniformityNormalized
lbp-3D-k_glszm_GrayLevelVariance
lbp-3D-k_glszm_HighGrayLevelZoneEmphasis
lbp-3D-k_glszm_LargeAreaEmphasis
lbp-3D-k_glszm_LargeAreaHighGrayLevelEmphasis
lbp-3D-k_glszm_LargeAreaLowGrayLevelEmphasis
lbp-3D-k_glszm_LowGrayLevelZoneEmphasis
lbp-3D-k_glszm_SizeZoneNonUniformity
lbp-3D-k_glszm_SizeZoneNonUniformityNormalized
lbp-3D-k_glszm_SmallAreaEmphasis
lbp-3D-k_glszm_SmallAreaHighGrayLevelEmphasis

lbp-3D-k_glszm_SmallAreaLowGrayLevelEmphasis
lbp-3D-k_glszm_ZoneEntropy
lbp-3D-k_glszm_ZonePercentage
lbp-3D-k_glszm_ZoneVariance
lbp-3D-k_ngtdm_Busyness
lbp-3D-k_ngtdm_Coarseness
lbp-3D-k_ngtdm_Complexity
lbp-3D-k_ngtdm_Contrast
lbp-3D-k_ngtdm_Strength
log-sigma-1-0-mm-3D_firstorder_10Percentile
log-sigma-1-0-mm-3D_firstorder_90Percentile
log-sigma-1-0-mm-3D_firstorder_Energy
log-sigma-1-0-mm-3D_firstorder_Entropy
log-sigma-1-0-mm-3D_firstorder_InterquartileRange
log-sigma-1-0-mm-3D_firstorder_Kurtosis
log-sigma-1-0-mm-3D_firstorder_Maximum
log-sigma-1-0-mm-3D_firstorder_MeanAbsoluteDeviation
log-sigma-1-0-mm-3D_firstorder_Mean
log-sigma-1-0-mm-3D_firstorder_Median
log-sigma-1-0-mm-3D_firstorder_Minimum
log-sigma-1-0-mm-3D_firstorder_Range
log-sigma-1-0-mm-3D_firstorder_RobustMeanAbsoluteDeviation
log-sigma-1-0-mm-3D_firstorder_RootMeanSquared
log-sigma-1-0-mm-3D_firstorder_Skewness
log-sigma-1-0-mm-3D_firstorder_TotalEnergy
log-sigma-1-0-mm-3D_firstorder_Uniformity
log-sigma-1-0-mm-3D_firstorder_Variance
log-sigma-1-0-mm-3D_glcm_Autocorrelation
log-sigma-1-0-mm-3D_glcm_ClusterProminence
log-sigma-1-0-mm-3D_glcm_ClusterShade
log-sigma-1-0-mm-3D_glcm_ClusterTendency
log-sigma-1-0-mm-3D_glcm_Contrast
log-sigma-1-0-mm-3D_glcm_Correlation
log-sigma-1-0-mm-3D_glcm_DifferenceAverage
log-sigma-1-0-mm-3D_glcm_DifferenceEntropy
log-sigma-1-0-mm-3D_glcm_DifferenceVariance
log-sigma-1-0-mm-3D_glcm_Id
log-sigma-1-0-mm-3D_glcm_Idm
log-sigma-1-0-mm-3D_glcm_Idmn
log-sigma-1-0-mm-3D_glcm_Idn
log-sigma-1-0-mm-3D_glcm_Imc1
log-sigma-1-0-mm-3D_glcm_Imc2
log-sigma-1-0-mm-3D_glcm_InverseVariance
log-sigma-1-0-mm-3D_glcm_JointAverage
log-sigma-1-0-mm-3D_glcm_JointEnergy
log-sigma-1-0-mm-3D_glcm_JointEntropy
log-sigma-1-0-mm-3D_glcm_MCC

log-sigma-1-0-mm-3D_glcm_MaximumProbability
log-sigma-1-0-mm-3D_glcm_SumAverage
log-sigma-1-0-mm-3D_glcm_SumEntropy
log-sigma-1-0-mm-3D_glcm_SumSquares
log-sigma-1-0-mm-3D_gldm_DependenceEntropy
log-sigma-1-0-mm-3D_gldm_DependenceNonUniformity
log-sigma-1-0-mm-3D_gldm_DependenceNonUniformityNormalized
log-sigma-1-0-mm-3D_gldm_DependenceVariance
log-sigma-1-0-mm-3D_gldm_GrayLevelNonUniformity
log-sigma-1-0-mm-3D_gldm_GrayLevelVariance
log-sigma-1-0-mm-3D_gldm_HighGrayLevelEmphasis
log-sigma-1-0-mm-3D_gldm_LargeDependenceEmphasis
log-sigma-1-0-mm-3D_gldm_LargeDependenceHighGrayLevelEmphasis
log-sigma-1-0-mm-3D_gldm_LargeDependenceLowGrayLevelEmphasis
log-sigma-1-0-mm-3D_gldm_LowGrayLevelEmphasis
log-sigma-1-0-mm-3D_gldm_SmallDependenceEmphasis
log-sigma-1-0-mm-3D_gldm_SmallDependenceHighGrayLevelEmphasis
log-sigma-1-0-mm-3D_gldm_SmallDependenceLowGrayLevelEmphasis
log-sigma-1-0-mm-3D_glrlm_GrayLevelNonUniformity
log-sigma-1-0-mm-3D_glrlm_GrayLevelNonUniformityNormalized
log-sigma-1-0-mm-3D_glrlm_GrayLevelVariance
log-sigma-1-0-mm-3D_glrlm_HighGrayLevelRunEmphasis
log-sigma-1-0-mm-3D_glrlm_LongRunEmphasis
log-sigma-1-0-mm-3D_glrlm_LongRunHighGrayLevelEmphasis
log-sigma-1-0-mm-3D_glrlm_LongRunLowGrayLevelEmphasis
log-sigma-1-0-mm-3D_glrlm_LowGrayLevelRunEmphasis
log-sigma-1-0-mm-3D_glrlm_RunEntropy
log-sigma-1-0-mm-3D_glrlm_RunLengthNonUniformity
log-sigma-1-0-mm-3D_glrlm_RunLengthNonUniformityNormalized
log-sigma-1-0-mm-3D_glrlm_RunPercentage
log-sigma-1-0-mm-3D_glrlm_RunVariance
log-sigma-1-0-mm-3D_glrlm_ShortRunEmphasis
log-sigma-1-0-mm-3D_glrlm_ShortRunHighGrayLevelEmphasis
log-sigma-1-0-mm-3D_glrlm_ShortRunLowGrayLevelEmphasis
log-sigma-1-0-mm-3D_glszm_GrayLevelNonUniformity
log-sigma-1-0-mm-3D_glszm_GrayLevelNonUniformityNormalized
log-sigma-1-0-mm-3D_glszm_GrayLevelVariance
log-sigma-1-0-mm-3D_glszm_HighGrayLevelZoneEmphasis
log-sigma-1-0-mm-3D_glszm_LargeAreaEmphasis
log-sigma-1-0-mm-3D_glszm_LargeAreaHighGrayLevelEmphasis
log-sigma-1-0-mm-3D_glszm_LargeAreaLowGrayLevelEmphasis
log-sigma-1-0-mm-3D_glszm_LowGrayLevelZoneEmphasis
log-sigma-1-0-mm-3D_glszm_SizeZoneNonUniformity
log-sigma-1-0-mm-3D_glszm_SizeZoneNonUniformityNormalized

log-sigma-1-0-mm-3D_glszm_SmallAreaEmphasis
log-sigma-1-0-mm-3D_glszm_SmallAreaHighGrayLevelEmphasis
log-sigma-1-0-mm-3D_glszm_SmallAreaLowGrayLevelEmphasis
log-sigma-1-0-mm-3D_glszm_ZoneEntropy
log-sigma-1-0-mm-3D_glszm_ZonePercentage
log-sigma-1-0-mm-3D_glszm_ZoneVariance
log-sigma-1-0-mm-3D_ngtdm_Busyness
log-sigma-1-0-mm-3D_ngtdm_Coarseness
log-sigma-1-0-mm-3D_ngtdm_Complexity
log-sigma-1-0-mm-3D_ngtdm_Contrast
log-sigma-1-0-mm-3D_ngtdm_Strength
log-sigma-2-0-mm-3D_firstorder_10Percentile
log-sigma-2-0-mm-3D_firstorder_90Percentile
log-sigma-2-0-mm-3D_firstorder_Energy
log-sigma-2-0-mm-3D_firstorder_Entropy
log-sigma-2-0-mm-3D_firstorder_InterquartileRange
log-sigma-2-0-mm-3D_firstorder_Kurtosis
log-sigma-2-0-mm-3D_firstorder_Maximum
log-sigma-2-0-mm-3D_firstorder_MeanAbsoluteDeviation
log-sigma-2-0-mm-3D_firstorder_Mean
log-sigma-2-0-mm-3D_firstorder_Median
log-sigma-2-0-mm-3D_firstorder_Minimum
log-sigma-2-0-mm-3D_firstorder_Range
log-sigma-2-0-mm-3D_firstorder_RobustMeanAbsoluteDeviation
log-sigma-2-0-mm-3D_firstorder_RootMeanSquared
log-sigma-2-0-mm-3D_firstorder_Skewness
log-sigma-2-0-mm-3D_firstorder_TotalEnergy
log-sigma-2-0-mm-3D_firstorder_Uniformity
log-sigma-2-0-mm-3D_firstorder_Variance
log-sigma-2-0-mm-3D_glcm_Autocorrelation
log-sigma-2-0-mm-3D_glcm_ClusterProminence
log-sigma-2-0-mm-3D_glcm_ClusterShade
log-sigma-2-0-mm-3D_glcm_ClusterTendency
log-sigma-2-0-mm-3D_glcm_Contrast
log-sigma-2-0-mm-3D_glcm_Correlation
log-sigma-2-0-mm-3D_glcm_DifferenceAverage
log-sigma-2-0-mm-3D_glcm_DifferenceEntropy
log-sigma-2-0-mm-3D_glcm_DifferenceVariance
log-sigma-2-0-mm-3D_glcm_Id
log-sigma-2-0-mm-3D_glcm_Idm
log-sigma-2-0-mm-3D_glcm_Idmn
log-sigma-2-0-mm-3D_glcm_Idn
log-sigma-2-0-mm-3D_glcm_Imc1
log-sigma-2-0-mm-3D_glcm_Imc2
log-sigma-2-0-mm-3D_glcm_InverseVariance
log-sigma-2-0-mm-3D_glcm_JointAverage
log-sigma-2-0-mm-3D_glcm_JointEnergy

log-sigma-2-0-mm-3D_glcm_JointEntropy
log-sigma-2-0-mm-3D_glcm_MCC
log-sigma-2-0-mm-3D_glcm_MaximumProbability
log-sigma-2-0-mm-3D_glcm_SumAverage
log-sigma-2-0-mm-3D_glcm_SumEntropy
log-sigma-2-0-mm-3D_glcm_SumSquares
log-sigma-2-0-mm-3D_gldm_DependenceEntropy
log-sigma-2-0-mm-3D_gldm_DependenceNonUniformity
log-sigma-2-0-mm-3D_gldm_DependenceNonUniformityNormalized
log-sigma-2-0-mm-3D_gldm_DependenceVariance
log-sigma-2-0-mm-3D_gldm_GrayLevelNonUniformity
log-sigma-2-0-mm-3D_gldm_GrayLevelVariance
log-sigma-2-0-mm-3D_gldm_HighGrayLevelEmphasis
log-sigma-2-0-mm-3D_gldm_LargeDependenceEmphasis
log-sigma-2-0-mm-3D_gldm_LargeDependenceHighGrayLevelEmphasis
log-sigma-2-0-mm-3D_gldm_LargeDependenceLowGrayLevelEmphasis
log-sigma-2-0-mm-3D_gldm_LowGrayLevelEmphasis
log-sigma-2-0-mm-3D_gldm_SmallDependenceEmphasis
log-sigma-2-0-mm-3D_gldm_SmallDependenceHighGrayLevelEmphasis
log-sigma-2-0-mm-3D_gldm_SmallDependenceLowGrayLevelEmphasis
log-sigma-2-0-mm-3D_glrlm_GrayLevelNonUniformity
log-sigma-2-0-mm-3D_glrlm_GrayLevelNonUniformityNormalized
log-sigma-2-0-mm-3D_glrlm_GrayLevelVariance
log-sigma-2-0-mm-3D_glrlm_HighGrayLevelRunEmphasis
log-sigma-2-0-mm-3D_glrlm_LongRunEmphasis
log-sigma-2-0-mm-3D_glrlm_LongRunHighGrayLevelEmphasis
log-sigma-2-0-mm-3D_glrlm_LongRunLowGrayLevelEmphasis
log-sigma-2-0-mm-3D_glrlm_LowGrayLevelRunEmphasis
log-sigma-2-0-mm-3D_glrlm_RunEntropy
log-sigma-2-0-mm-3D_glrlm_RunLengthNonUniformity
log-sigma-2-0-mm-3D_glrlm_RunLengthNonUniformityNormalized
log-sigma-2-0-mm-3D_glrlm_RunPercentage
log-sigma-2-0-mm-3D_glrlm_RunVariance
log-sigma-2-0-mm-3D_glrlm_ShortRunEmphasis
log-sigma-2-0-mm-3D_glrlm_ShortRunHighGrayLevelEmphasis
log-sigma-2-0-mm-3D_glrlm_ShortRunLowGrayLevelEmphasis
log-sigma-2-0-mm-3D_glszm_GrayLevelNonUniformity
log-sigma-2-0-mm-3D_glszm_GrayLevelNonUniformityNormalized
log-sigma-2-0-mm-3D_glszm_GrayLevelVariance
log-sigma-2-0-mm-3D_glszm_HighGrayLevelZoneEmphasis
log-sigma-2-0-mm-3D_glszm_LargeAreaEmphasis
log-sigma-2-0-mm-3D_glszm_LargeAreaHighGrayLevelEmphasis
log-sigma-2-0-mm-3D_glszm_LargeAreaLowGrayLevelEmphasis
log-sigma-2-0-mm-3D_glszm_LowGrayLevelZoneEmphasis

log-sigma-2-0-mm-3D_glszm_SizeZoneNonUniformity
log-sigma-2-0-mm-3D_glszm_SizeZoneNonUniformityNormalized
log-sigma-2-0-mm-3D_glszm_SmallAreaEmphasis
log-sigma-2-0-mm-3D_glszm_SmallAreaHighGrayLevelEmphasis
log-sigma-2-0-mm-3D_glszm_SmallAreaLowGrayLevelEmphasis
log-sigma-2-0-mm-3D_glszm_ZoneEntropy
log-sigma-2-0-mm-3D_glszm_ZonePercentage
log-sigma-2-0-mm-3D_glszm_ZoneVariance
log-sigma-2-0-mm-3D_ngtdm_Busyness
log-sigma-2-0-mm-3D_ngtdm_Coarseness
log-sigma-2-0-mm-3D_ngtdm_Complexity
log-sigma-2-0-mm-3D_ngtdm_Contrast
log-sigma-2-0-mm-3D_ngtdm_Strength
log-sigma-5-0-mm-3D_firstorder_10Percentile
log-sigma-5-0-mm-3D_firstorder_90Percentile
log-sigma-5-0-mm-3D_firstorder_Energy
log-sigma-5-0-mm-3D_firstorder_Entropy
log-sigma-5-0-mm-3D_firstorder_InterquartileRange
log-sigma-5-0-mm-3D_firstorder_Kurtosis
log-sigma-5-0-mm-3D_firstorder_Maximum
log-sigma-5-0-mm-3D_firstorder_MeanAbsoluteDeviation
log-sigma-5-0-mm-3D_firstorder_Mean
log-sigma-5-0-mm-3D_firstorder_Median
log-sigma-5-0-mm-3D_firstorder_Minimum
log-sigma-5-0-mm-3D_firstorder_Range
log-sigma-5-0-mm-3D_firstorder_RobustMeanAbsoluteDeviation
log-sigma-5-0-mm-3D_firstorder_RootMeanSquared
log-sigma-5-0-mm-3D_firstorder_Skewness
log-sigma-5-0-mm-3D_firstorder_TotalEnergy
log-sigma-5-0-mm-3D_firstorder_Uniformity
log-sigma-5-0-mm-3D_firstorder_Variance
log-sigma-5-0-mm-3D_glcm_Autocorrelation
log-sigma-5-0-mm-3D_glcm_ClusterProminence
log-sigma-5-0-mm-3D_glcm_ClusterShade
log-sigma-5-0-mm-3D_glcm_ClusterTendency
log-sigma-5-0-mm-3D_glcm_Contrast
log-sigma-5-0-mm-3D_glcm_Correlation
log-sigma-5-0-mm-3D_glcm_DifferenceAverage
log-sigma-5-0-mm-3D_glcm_DifferenceEntropy
log-sigma-5-0-mm-3D_glcm_DifferenceVariance
log-sigma-5-0-mm-3D_glcm_Id
log-sigma-5-0-mm-3D_glcm_Idm
log-sigma-5-0-mm-3D_glcm_Idmn
log-sigma-5-0-mm-3D_glcm_Idn
log-sigma-5-0-mm-3D_glcm_Imc1
log-sigma-5-0-mm-3D_glcm_Imc2
log-sigma-5-0-mm-3D_glcm_InverseVariance

log-sigma-5-0-mm-3D_glcm_JointAverage
log-sigma-5-0-mm-3D_glcm_JointEnergy
log-sigma-5-0-mm-3D_glcm_JointEntropy
log-sigma-5-0-mm-3D_glcm_MCC
log-sigma-5-0-mm-3D_glcm_MaximumProbability
log-sigma-5-0-mm-3D_glcm_SumAverage
log-sigma-5-0-mm-3D_glcm_SumEntropy
log-sigma-5-0-mm-3D_glcm_SumSquares
log-sigma-5-0-mm-3D_gldm_DependenceEntropy
log-sigma-5-0-mm-3D_gldm_DependenceNonUniformity
log-sigma-5-0-mm-3D_gldm_DependenceNonUniformityNormalized
log-sigma-5-0-mm-3D_gldm_DependenceVariance
log-sigma-5-0-mm-3D_gldm_GrayLevelNonUniformity
log-sigma-5-0-mm-3D_gldm_GrayLevelVariance
log-sigma-5-0-mm-3D_gldm_HighGrayLevelEmphasis
log-sigma-5-0-mm-3D_gldm_LargeDependenceEmphasis
log-sigma-5-0-mm-3D_gldm_LargeDependenceHighGrayLevelEmphasis
log-sigma-5-0-mm-3D_gldm_LargeDependenceLowGrayLevelEmphasis
log-sigma-5-0-mm-3D_gldm_LowGrayLevelEmphasis
log-sigma-5-0-mm-3D_gldm_SmallDependenceEmphasis
log-sigma-5-0-mm-3D_gldm_SmallDependenceHighGrayLevelEmphasis
log-sigma-5-0-mm-3D_gldm_SmallDependenceLowGrayLevelEmphasis
log-sigma-5-0-mm-3D_glrlm_GrayLevelNonUniformity
log-sigma-5-0-mm-3D_glrlm_GrayLevelNonUniformityNormalized
log-sigma-5-0-mm-3D_glrlm_GrayLevelVariance
log-sigma-5-0-mm-3D_glrlm_HighGrayLevelRunEmphasis
log-sigma-5-0-mm-3D_glrlm_LongRunEmphasis
log-sigma-5-0-mm-3D_glrlm_LongRunHighGrayLevelEmphasis
log-sigma-5-0-mm-3D_glrlm_LongRunLowGrayLevelEmphasis
log-sigma-5-0-mm-3D_glrlm_LowGrayLevelRunEmphasis
log-sigma-5-0-mm-3D_glrlm_RunEntropy
log-sigma-5-0-mm-3D_glrlm_RunLengthNonUniformity
log-sigma-5-0-mm-3D_glrlm_RunLengthNonUniformityNormalized
log-sigma-5-0-mm-3D_glrlm_RunPercentage
log-sigma-5-0-mm-3D_glrlm_RunVariance
log-sigma-5-0-mm-3D_glrlm_ShortRunEmphasis
log-sigma-5-0-mm-3D_glrlm_ShortRunHighGrayLevelEmphasis
log-sigma-5-0-mm-3D_glrlm_ShortRunLowGrayLevelEmphasis
log-sigma-5-0-mm-3D_glszm_GrayLevelNonUniformity
log-sigma-5-0-mm-3D_glszm_GrayLevelNonUniformityNormalized
log-sigma-5-0-mm-3D_glszm_GrayLevelVariance
log-sigma-5-0-mm-3D_glszm_HighGrayLevelZoneEmphasis
log-sigma-5-0-mm-3D_glszm_LargeAreaEmphasis
log-sigma-5-0-mm-3D_glszm_LargeAreaHighGrayLevelEmphasis

log-sigma-5-0-mm-3D_glszm_LargeAreaLowGrayLevelEmphasis
log-sigma-5-0-mm-3D_glszm_LowGrayLevelZoneEmphasis
log-sigma-5-0-mm-3D_glszm_SizeZoneNonUniformity
log-sigma-5-0-mm-3D_glszm_SizeZoneNonUniformityNormalized
log-sigma-5-0-mm-3D_glszm_SmallAreaEmphasis
log-sigma-5-0-mm-3D_glszm_SmallAreaHighGrayLevelEmphasis
log-sigma-5-0-mm-3D_glszm_SmallAreaLowGrayLevelEmphasis
log-sigma-5-0-mm-3D_glszm_ZoneEntropy
log-sigma-5-0-mm-3D_glszm_ZonePercentage
log-sigma-5-0-mm-3D_glszm_ZoneVariance
log-sigma-5-0-mm-3D_ngtdm_Busyness
log-sigma-5-0-mm-3D_ngtdm_Coarseness
log-sigma-5-0-mm-3D_ngtdm_Complexity
log-sigma-5-0-mm-3D_ngtdm_Contrast
log-sigma-5-0-mm-3D_ngtdm_Strength
logarithm_firstorder_10Percentile
logarithm_firstorder_90Percentile
logarithm_firstorder_Energy
logarithm_firstorder_Entropy
logarithm_firstorder_InterquartileRange
logarithm_firstorder_Kurtosis
logarithm_firstorder_Maximum
logarithm_firstorder_MeanAbsoluteDeviation
logarithm_firstorder_Mean
logarithm_firstorder_Median
logarithm_firstorder_Minimum
logarithm_firstorder_Range
logarithm_firstorder_RobustMeanAbsoluteDeviation
logarithm_firstorder_RootMeanSquared
logarithm_firstorder_Skewness
logarithm_firstorder_TotalEnergy
logarithm_firstorder_Uniformity
logarithm_firstorder_Variance
logarithm_glcm_Autocorrelation
logarithm_glcm_ClusterProminence
logarithm_glcm_ClusterShade
logarithm_glcm_ClusterTendency
logarithm_glcm_Contrast
logarithm_glcm_Correlation
logarithm_glcm_DifferenceAverage
logarithm_glcm_DifferenceEntropy
logarithm_glcm_DifferenceVariance
logarithm_glcm_Id
logarithm_glcm_Idm
logarithm_glcm_Idmn
logarithm_glcm_Idn
logarithm_glcm_Imc1

logarithm_glcm_Imc2
logarithm_glcm_InverseVariance
logarithm_glcm_JointAverage
logarithm_glcm_JointEnergy
logarithm_glcm_JointEntropy
logarithm_glcm_MCC
logarithm_glcm_MaximumProbability
logarithm_glcm_SumAverage
logarithm_glcm_SumEntropy
logarithm_glcm_SumSquares
logarithm_gldm_DependenceEntropy
logarithm_gldm_DependenceNonUniformity
logarithm_gldm_DependenceNonUniformityNormalized
logarithm_gldm_DependenceVariance
logarithm_gldm_GrayLevelNonUniformity
logarithm_gldm_GrayLevelVariance
logarithm_gldm_HighGrayLevelEmphasis
logarithm_gldm_LargeDependenceEmphasis
logarithm_gldm_LargeDependenceHighGrayLevelEmphasis
logarithm_gldm_LargeDependenceLowGrayLevelEmphasis
logarithm_gldm_LowGrayLevelEmphasis
logarithm_gldm_SmallDependenceEmphasis
logarithm_gldm_SmallDependenceHighGrayLevelEmphasis
logarithm_gldm_SmallDependenceLowGrayLevelEmphasis
logarithm_glrlm_GrayLevelNonUniformity
logarithm_glrlm_GrayLevelNonUniformityNormalized
logarithm_glrlm_GrayLevelVariance
logarithm_glrlm_HighGrayLevelRunEmphasis
logarithm_glrlm_LongRunEmphasis
logarithm_glrlm_LongRunHighGrayLevelEmphasis
logarithm_glrlm_LongRunLowGrayLevelEmphasis
logarithm_glrlm_LowGrayLevelRunEmphasis
logarithm_glrlm_RunEntropy
logarithm_glrlm_RunLengthNonUniformity
logarithm_glrlm_RunLengthNonUniformityNormalized
logarithm_glrlm_RunPercentage
logarithm_glrlm_RunVariance
logarithm_glrlm_ShortRunEmphasis
logarithm_glrlm_ShortRunHighGrayLevelEmphasis
logarithm_glrlm_ShortRunLowGrayLevelEmphasis
logarithm_glszm_GrayLevelNonUniformity
logarithm_glszm_GrayLevelNonUniformityNormalized
logarithm_glszm_GrayLevelVariance
logarithm_glszm_HighGrayLevelZoneEmphasis
logarithm_glszm_LargeAreaEmphasis
logarithm_glszm_LargeAreaHighGrayLevelEmphasis
logarithm_glszm_LargeAreaLowGrayLevelEmphasis

logarithm_glszm_LowGrayLevelZoneEmphasis
logarithm_glszm_SizeZoneNonUniformity
logarithm_glszm_SizeZoneNonUniformityNormalized
logarithm_glszm_SmallAreaEmphasis
logarithm_glszm_SmallAreaHighGrayLevelEmphasis
logarithm_glszm_SmallAreaLowGrayLevelEmphasis
logarithm_glszm_ZoneEntropy
logarithm_glszm_ZonePercentage
logarithm_glszm_ZoneVariance
logarithm_ngtdm_Busyness
logarithm_ngtdm_Coarseness
logarithm_ngtdm_Complexity
logarithm_ngtdm_Contrast
logarithm_ngtdm_Strength
square_firstorder_10Percentile
square_firstorder_90Percentile
square_firstorder_Energy
square_firstorder_Entropy
square_firstorder_InterquartileRange
square_firstorder_Kurtosis
square_firstorder_Maximum
square_firstorder_MeanAbsoluteDeviation
square_firstorder_Mean
square_firstorder_Median
square_firstorder_Minimum
square_firstorder_Range
square_firstorder_RobustMeanAbsoluteDeviation
square_firstorder_RootMeanSquared
square_firstorder_Skewness
square_firstorder_TotalEnergy
square_firstorder_Uniformity
square_firstorder_Variance
square_glcm_Autocorrelation
square_glcm_ClusterProminence
square_glcm_ClusterShade
square_glcm_ClusterTendency
square_glcm_Contrast
square_glcm_Correlation
square_glcm_DifferenceAverage
square_glcm_DifferenceEntropy
square_glcm_DifferenceVariance
square_glcm_Id
square_glcm_Idm
square_glcm_Idmn
square_glcm_Idn
square_glcm_Imc1
square_glcm_Imc2

square_glcm_InverseVariance
square_glcm_JointAverage
square_glcm_JointEnergy
square_glcm_JointEntropy
square_glcm_MCC
square_glcm_MaximumProbability
square_glcm_SumAverage
square_glcm_SumEntropy
square_glcm_SumSquares
square_gldm_DependenceEntropy
square_gldm_DependenceNonUniformity
square_gldm_DependenceNonUniformityNormalized
square_gldm_DependenceVariance
square_gldm_GrayLevelNonUniformity
square_gldm_GrayLevelVariance
square_gldm_HighGrayLevelEmphasis
square_gldm_LargeDependenceEmphasis
square_gldm_LargeDependenceHighGrayLevelEmphasis
square_gldm_LargeDependenceLowGrayLevelEmphasis
square_gldm_LowGrayLevelEmphasis
square_gldm_SmallDependenceEmphasis
square_gldm_SmallDependenceHighGrayLevelEmphasis
square_gldm_SmallDependenceLowGrayLevelEmphasis
square_glrlm_GrayLevelNonUniformity
square_glrlm_GrayLevelNonUniformityNormalized
square_glrlm_GrayLevelVariance
square_glrlm_HighGrayLevelRunEmphasis
square_glrlm_LongRunEmphasis
square_glrlm_LongRunHighGrayLevelEmphasis
square_glrlm_LongRunLowGrayLevelEmphasis
square_glrlm_LowGrayLevelRunEmphasis
square_glrlm_RunEntropy
square_glrlm_RunLengthNonUniformity
square_glrlm_RunLengthNonUniformityNormalized
square_glrlm_RunPercentage
square_glrlm_RunVariance
square_glrlm_ShortRunEmphasis
square_glrlm_ShortRunHighGrayLevelEmphasis
square_glrlm_ShortRunLowGrayLevelEmphasis
square_glszm_GrayLevelNonUniformity
square_glszm_GrayLevelNonUniformityNormalized
square_glszm_GrayLevelVariance
square_glszm_HighGrayLevelZoneEmphasis
square_glszm_LargeAreaEmphasis
square_glszm_LargeAreaHighGrayLevelEmphasis
square_glszm_LargeAreaLowGrayLevelEmphasis
square_glszm_LowGrayLevelZoneEmphasis

square_glszm_SizeZoneNonUniformity
square_glszm_SizeZoneNonUniformityNormalized
square_glszm_SmallAreaEmphasis
square_glszm_SmallAreaHighGrayLevelEmphasis
square_glszm_SmallAreaLowGrayLevelEmphasis
square_glszm_ZoneEntropy
square_glszm_ZonePercentage
square_glszm_ZoneVariance
square_ngtdm_Busyness
square_ngtdm_Coarseness
square_ngtdm_Complexity
square_ngtdm_Contrast
square_ngtdm_Strength
squareroot_firstorder_10Percentile
squareroot_firstorder_90Percentile
squareroot_firstorder_Energy
squareroot_firstorder_Entropy
squareroot_firstorder_InterquartileRange
squareroot_firstorder_Kurtosis
squareroot_firstorder_Maximum
squareroot_firstorder_MeanAbsoluteDeviation
squareroot_firstorder_Mean
squareroot_firstorder_Median
squareroot_firstorder_Minimum
squareroot_firstorder_Range
squareroot_firstorder_RobustMeanAbsoluteDeviation
squareroot_firstorder_RootMeanSquared
squareroot_firstorder_Skewness
squareroot_firstorder_TotalEnergy
squareroot_firstorder_Uniformity
squareroot_firstorder_Variance
squareroot_glcm_Autocorrelation
squareroot_glcm_ClusterProminence
squareroot_glcm_ClusterShade
squareroot_glcm_ClusterTendency
squareroot_glcm_Contrast
squareroot_glcm_Correlation
squareroot_glcm_DifferenceAverage
squareroot_glcm_DifferenceEntropy
squareroot_glcm_DifferenceVariance
squareroot_glcm_Id
squareroot_glcm_Idm
squareroot_glcm_Idmn
squareroot_glcm_Idn
squareroot_glcm_Imc1
squareroot_glcm_Imc2
squareroot_glcm_InverseVariance

squareroot_glcm_JointAverage
squareroot_glcm_JointEnergy
squareroot_glcm_JointEntropy
squareroot_glcm_MCC
squareroot_glcm_MaximumProbability
squareroot_glcm_SumAverage
squareroot_glcm_SumEntropy
squareroot_glcm_SumSquares
squareroot_gldm_DependenceEntropy
squareroot_gldm_DependenceNonUniformity
squareroot_gldm_DependenceNonUniformityNormalized
squareroot_gldm_DependenceVariance
squareroot_gldm_GrayLevelNonUniformity
squareroot_gldm_GrayLevelVariance
squareroot_gldm_HighGrayLevelEmphasis
squareroot_gldm_LargeDependenceEmphasis
squareroot_gldm_LargeDependenceHighGrayLevelEmphasis
squareroot_gldm_LargeDependenceLowGrayLevelEmphasis
squareroot_gldm_LowGrayLevelEmphasis
squareroot_gldm_SmallDependenceEmphasis
squareroot_gldm_SmallDependenceHighGrayLevelEmphasis
squareroot_gldm_SmallDependenceLowGrayLevelEmphasis
squareroot_glrlm_GrayLevelNonUniformity
squareroot_glrlm_GrayLevelNonUniformityNormalized
squareroot_glrlm_GrayLevelVariance
squareroot_glrlm_HighGrayLevelRunEmphasis
squareroot_glrlm_LongRunEmphasis
squareroot_glrlm_LongRunHighGrayLevelEmphasis
squareroot_glrlm_LongRunLowGrayLevelEmphasis
squareroot_glrlm_LowGrayLevelRunEmphasis
squareroot_glrlm_RunEntropy
squareroot_glrlm_RunLengthNonUniformity
squareroot_glrlm_RunLengthNonUniformityNormalized
squareroot_glrlm_RunPercentage
squareroot_glrlm_RunVariance
squareroot_glrlm_ShortRunEmphasis
squareroot_glrlm_ShortRunHighGrayLevelEmphasis
squareroot_glrlm_ShortRunLowGrayLevelEmphasis
squareroot_glszm_GrayLevelNonUniformity
squareroot_glszm_GrayLevelNonUniformityNormalized
squareroot_glszm_GrayLevelVariance
squareroot_glszm_HighGrayLevelZoneEmphasis
squareroot_glszm_LargeAreaEmphasis
squareroot_glszm_LargeAreaHighGrayLevelEmphasis
squareroot_glszm_LargeAreaLowGrayLevelEmphasis
squareroot_glszm_LowGrayLevelZoneEmphasis
squareroot_glszm_SizeZoneNonUniformity

squareroot_glszm_SizeZoneNonUniformityNormalized
squareroot_glszm_SmallAreaEmphasis
squareroot_glszm_SmallAreaHighGrayLevelEmphasis
squareroot_glszm_SmallAreaLowGrayLevelEmphasis
squareroot_glszm_ZoneEntropy
squareroot_glszm_ZonePercentage
squareroot_glszm_ZoneVariance
squareroot_ngtdm_Busyness
squareroot_ngtdm_Coarseness
squareroot_ngtdm_Complexity
squareroot_ngtdm_Contrast
squareroot_ngtdm_Strength
wavelet-LLH_firstorder_10Percentile
wavelet-LLH_firstorder_90Percentile
wavelet-LLH_firstorder_Energy
wavelet-LLH_firstorder_Entropy
wavelet-LLH_firstorder_InterquartileRange
wavelet-LLH_firstorder_Kurtosis
wavelet-LLH_firstorder_Maximum
wavelet-LLH_firstorder_MeanAbsoluteDeviation
wavelet-LLH_firstorder_Mean
wavelet-LLH_firstorder_Median
wavelet-LLH_firstorder_Minimum
wavelet-LLH_firstorder_Range
wavelet-LLH_firstorder_RobustMeanAbsoluteDeviation
wavelet-LLH_firstorder_RootMeanSquared
wavelet-LLH_firstorder_Skewness
wavelet-LLH_firstorder_TotalEnergy
wavelet-LLH_firstorder_Uniformity
wavelet-LLH_firstorder_Variance
wavelet-LLH_glcm_Autocorrelation
wavelet-LLH_glcm_ClusterProminence
wavelet-LLH_glcm_ClusterShade
wavelet-LLH_glcm_ClusterTendency
wavelet-LLH_glcm_Contrast
wavelet-LLH_glcm_Correlation
wavelet-LLH_glcm_DifferenceAverage
wavelet-LLH_glcm_DifferenceEntropy
wavelet-LLH_glcm_DifferenceVariance
wavelet-LLH_glcm_Id
wavelet-LLH_glcm_Idm
wavelet-LLH_glcm_Idmn
wavelet-LLH_glcm_Idn
wavelet-LLH_glcm_Imc1
wavelet-LLH_glcm_Imc2
wavelet-LLH_glcm_InverseVariance
wavelet-LLH_glcm_JointAverage

wavelet-LLH_glcm_JointEnergy
wavelet-LLH_glcm_JointEntropy
wavelet-LLH_glcm_MCC
wavelet-LLH_glcm_MaximumProbability
wavelet-LLH_glcm_SumAverage
wavelet-LLH_glcm_SumEntropy
wavelet-LLH_glcm_SumSquares
wavelet-LLH_gldm_DependenceEntropy
wavelet-LLH_gldm_DependenceNonUniformity
wavelet-LLH_gldm_DependenceNonUniformityNormalized
wavelet-LLH_gldm_DependenceVariance
wavelet-LLH_gldm_GrayLevelNonUniformity
wavelet-LLH_gldm_GrayLevelVariance
wavelet-LLH_gldm_HighGrayLevelEmphasis
wavelet-LLH_gldm_LargeDependenceEmphasis
wavelet-LLH_gldm_LargeDependenceHighGrayLevelEmphasis
wavelet-LLH_gldm_LargeDependenceLowGrayLevelEmphasis
wavelet-LLH_gldm_LowGrayLevelEmphasis
wavelet-LLH_gldm_SmallDependenceEmphasis
wavelet-LLH_gldm_SmallDependenceHighGrayLevelEmphasis
wavelet-LLH_gldm_SmallDependenceLowGrayLevelEmphasis
wavelet-LLH_glrlm_GrayLevelNonUniformity
wavelet-LLH_glrlm_GrayLevelNonUniformityNormalized
wavelet-LLH_glrlm_GrayLevelVariance
wavelet-LLH_glrlm_HighGrayLevelRunEmphasis
wavelet-LLH_glrlm_LongRunEmphasis
wavelet-LLH_glrlm_LongRunHighGrayLevelEmphasis
wavelet-LLH_glrlm_LongRunLowGrayLevelEmphasis
wavelet-LLH_glrlm_LowGrayLevelRunEmphasis
wavelet-LLH_glrlm_RunEntropy
wavelet-LLH_glrlm_RunLengthNonUniformity
wavelet-LLH_glrlm_RunLengthNonUniformityNormalized
wavelet-LLH_glrlm_RunPercentage
wavelet-LLH_glrlm_RunVariance
wavelet-LLH_glrlm_ShortRunEmphasis
wavelet-LLH_glrlm_ShortRunHighGrayLevelEmphasis
wavelet-LLH_glrlm_ShortRunLowGrayLevelEmphasis
wavelet-LLH_glszm_GrayLevelNonUniformity
wavelet-LLH_glszm_GrayLevelNonUniformityNormalized
wavelet-LLH_glszm_GrayLevelVariance
wavelet-LLH_glszm_HighGrayLevelZoneEmphasis
wavelet-LLH_glszm_LargeAreaEmphasis
wavelet-LLH_glszm_LargeAreaHighGrayLevelEmphasis
wavelet-LLH_glszm_LargeAreaLowGrayLevelEmphasis
wavelet-LLH_glszm_LowGrayLevelZoneEmphasis
wavelet-LLH_glszm_SizeZoneNonUniformity
wavelet-LLH_glszm_SizeZoneNonUniformityNormalized

wavelet-LLH_glszm_SmallAreaEmphasis
wavelet-LLH_glszm_SmallAreaHighGrayLevelEmphasis
wavelet-LLH_glszm_SmallAreaLowGrayLevelEmphasis
wavelet-LLH_glszm_ZoneEntropy
wavelet-LLH_glszm_ZonePercentage
wavelet-LLH_glszm_ZoneVariance
wavelet-LLH_ngtdm_Busyness
wavelet-LLH_ngtdm_Coarseness
wavelet-LLH_ngtdm_Complexity
wavelet-LLH_ngtdm_Contrast
wavelet-LLH_ngtdm_Strength
wavelet-LHL_firstorder_10Percentile
wavelet-LHL_firstorder_90Percentile
wavelet-LHL_firstorder_Energy
wavelet-LHL_firstorder_Entropy
wavelet-LHL_firstorder_InterquartileRange
wavelet-LHL_firstorder_Kurtosis
wavelet-LHL_firstorder_Maximum
wavelet-LHL_firstorder_MeanAbsoluteDeviation
wavelet-LHL_firstorder_Mean
wavelet-LHL_firstorder_Median
wavelet-LHL_firstorder_Minimum
wavelet-LHL_firstorder_Range
wavelet-LHL_firstorder_RobustMeanAbsoluteDeviation
wavelet-LHL_firstorder_RootMeanSquared
wavelet-LHL_firstorder_Skewness
wavelet-LHL_firstorder_TotalEnergy
wavelet-LHL_firstorder_Uniformity
wavelet-LHL_firstorder_Variance
wavelet-LHL_glcm_Autocorrelation
wavelet-LHL_glcm_ClusterProminence
wavelet-LHL_glcm_ClusterShade
wavelet-LHL_glcm_ClusterTendency
wavelet-LHL_glcm_Contrast
wavelet-LHL_glcm_Correlation
wavelet-LHL_glcm_DifferenceAverage
wavelet-LHL_glcm_DifferenceEntropy
wavelet-LHL_glcm_DifferenceVariance
wavelet-LHL_glcm_Id
wavelet-LHL_glcm_Idm
wavelet-LHL_glcm_Idmn
wavelet-LHL_glcm_Idn
wavelet-LHL_glcm_Imc1
wavelet-LHL_glcm_Imc2
wavelet-LHL_glcm_InverseVariance
wavelet-LHL_glcm_JointAverage
wavelet-LHL_glcm_JointEnergy

wavelet-LHL_glcm_JointEntropy
wavelet-LHL_glcm_MCC
wavelet-LHL_glcm_MaximumProbability
wavelet-LHL_glcm_SumAverage
wavelet-LHL_glcm_SumEntropy
wavelet-LHL_glcm_SumSquares
wavelet-LHL_gldm_DependenceEntropy
wavelet-LHL_gldm_DependenceNonUniformity
wavelet-LHL_gldm_DependenceNonUniformityNormalized
wavelet-LHL_gldm_DependenceVariance
wavelet-LHL_gldm_GrayLevelNonUniformity
wavelet-LHL_gldm_GrayLevelVariance
wavelet-LHL_gldm_HighGrayLevelEmphasis
wavelet-LHL_gldm_LargeDependenceEmphasis
wavelet-LHL_gldm_LargeDependenceHighGrayLevelEmphasis
wavelet-LHL_gldm_LargeDependenceLowGrayLevelEmphasis
wavelet-LHL_gldm_LowGrayLevelEmphasis
wavelet-LHL_gldm_SmallDependenceEmphasis
wavelet-LHL_gldm_SmallDependenceHighGrayLevelEmphasis
wavelet-LHL_gldm_SmallDependenceLowGrayLevelEmphasis
wavelet-LHL_glrlm_GrayLevelNonUniformity
wavelet-LHL_glrlm_GrayLevelNonUniformityNormalized
wavelet-LHL_glrlm_GrayLevelVariance
wavelet-LHL_glrlm_HighGrayLevelRunEmphasis
wavelet-LHL_glrlm_LongRunEmphasis
wavelet-LHL_glrlm_LongRunHighGrayLevelEmphasis
wavelet-LHL_glrlm_LongRunLowGrayLevelEmphasis
wavelet-LHL_glrlm_LowGrayLevelRunEmphasis
wavelet-LHL_glrlm_RunEntropy
wavelet-LHL_glrlm_RunLengthNonUniformity
wavelet-LHL_glrlm_RunLengthNonUniformityNormalized
wavelet-LHL_glrlm_RunPercentage
wavelet-LHL_glrlm_RunVariance
wavelet-LHL_glrlm_ShortRunEmphasis
wavelet-LHL_glrlm_ShortRunHighGrayLevelEmphasis
wavelet-LHL_glrlm_ShortRunLowGrayLevelEmphasis
wavelet-LHL_glszm_GrayLevelNonUniformity
wavelet-LHL_glszm_GrayLevelNonUniformityNormalized
wavelet-LHL_glszm_GrayLevelVariance
wavelet-LHL_glszm_HighGrayLevelZoneEmphasis
wavelet-LHL_glszm_LargeAreaEmphasis
wavelet-LHL_glszm_LargeAreaHighGrayLevelEmphasis
wavelet-LHL_glszm_LargeAreaLowGrayLevelEmphasis
wavelet-LHL_glszm_LowGrayLevelZoneEmphasis
wavelet-LHL_glszm_SizeZoneNonUniformity
wavelet-LHL_glszm_SizeZoneNonUniformityNormalized
wavelet-LHL_glszm_SmallAreaEmphasis

wavelet-LHL_glszm_SmallAreaHighGrayLevelEmphasis
wavelet-LHL_glszm_SmallAreaLowGrayLevelEmphasis
wavelet-LHL_glszm_ZoneEntropy
wavelet-LHL_glszm_ZonePercentage
wavelet-LHL_glszm_ZoneVariance
wavelet-LHL_ngtdm_Busyness
wavelet-LHL_ngtdm_Coarseness
wavelet-LHL_ngtdm_Complexity
wavelet-LHL_ngtdm_Contrast
wavelet-LHL_ngtdm_Strength
wavelet-LHH_firstorder_10Percentile
wavelet-LHH_firstorder_90Percentile
wavelet-LHH_firstorder_Energy
wavelet-LHH_firstorder_Entropy
wavelet-LHH_firstorder_InterquartileRange
wavelet-LHH_firstorder_Kurtosis
wavelet-LHH_firstorder_Maximum
wavelet-LHH_firstorder_MeanAbsoluteDeviation
wavelet-LHH_firstorder_Mean
wavelet-LHH_firstorder_Median
wavelet-LHH_firstorder_Minimum
wavelet-LHH_firstorder_Range
wavelet-LHH_firstorder_RobustMeanAbsoluteDeviation
wavelet-LHH_firstorder_RootMeanSquared
wavelet-LHH_firstorder_Skewness
wavelet-LHH_firstorder_TotalEnergy
wavelet-LHH_firstorder_Uniformity
wavelet-LHH_firstorder_Variance
wavelet-LHH_glcm_Autocorrelation
wavelet-LHH_glcm_ClusterProminence
wavelet-LHH_glcm_ClusterShade
wavelet-LHH_glcm_ClusterTendency
wavelet-LHH_glcm_Contrast
wavelet-LHH_glcm_Correlation
wavelet-LHH_glcm_DifferenceAverage
wavelet-LHH_glcm_DifferenceEntropy
wavelet-LHH_glcm_DifferenceVariance
wavelet-LHH_glcm_Id
wavelet-LHH_glcm_Idm
wavelet-LHH_glcm_Idmn
wavelet-LHH_glcm_Idn
wavelet-LHH_glcm_Imc1
wavelet-LHH_glcm_Imc2
wavelet-LHH_glcm_InverseVariance
wavelet-LHH_glcm_JointAverage
wavelet-LHH_glcm_JointEnergy
wavelet-LHH_glcm_JointEntropy

wavelet-LHH_glcm_MCC
wavelet-LHH_glcm_MaximumProbability
wavelet-LHH_glcm_SumAverage
wavelet-LHH_glcm_SumEntropy
wavelet-LHH_glcm_SumSquares
wavelet-LHH_gldm_DependenceEntropy
wavelet-LHH_gldm_DependenceNonUniformity
wavelet-LHH_gldm_DependenceNonUniformityNormalized
wavelet-LHH_gldm_DependenceVariance
wavelet-LHH_gldm_GrayLevelNonUniformity
wavelet-LHH_gldm_GrayLevelVariance
wavelet-LHH_gldm_HighGrayLevelEmphasis
wavelet-LHH_gldm_LargeDependenceEmphasis
wavelet-LHH_gldm_LargeDependenceHighGrayLevelEmphasis
wavelet-LHH_gldm_LargeDependenceLowGrayLevelEmphasis
wavelet-LHH_gldm_LowGrayLevelEmphasis
wavelet-LHH_gldm_SmallDependenceEmphasis
wavelet-LHH_gldm_SmallDependenceHighGrayLevelEmphasis
wavelet-LHH_gldm_SmallDependenceLowGrayLevelEmphasis
wavelet-LHH_glrlm_GrayLevelNonUniformity
wavelet-LHH_glrlm_GrayLevelNonUniformityNormalized
wavelet-LHH_glrlm_GrayLevelVariance
wavelet-LHH_glrlm_HighGrayLevelRunEmphasis
wavelet-LHH_glrlm_LongRunEmphasis
wavelet-LHH_glrlm_LongRunHighGrayLevelEmphasis
wavelet-LHH_glrlm_LongRunLowGrayLevelEmphasis
wavelet-LHH_glrlm_LowGrayLevelRunEmphasis
wavelet-LHH_glrlm_RunEntropy
wavelet-LHH_glrlm_RunLengthNonUniformity
wavelet-LHH_glrlm_RunLengthNonUniformityNormalized
wavelet-LHH_glrlm_RunPercentage
wavelet-LHH_glrlm_RunVariance
wavelet-LHH_glrlm_ShortRunEmphasis
wavelet-LHH_glrlm_ShortRunHighGrayLevelEmphasis
wavelet-LHH_glrlm_ShortRunLowGrayLevelEmphasis
wavelet-LHH_glszm_GrayLevelNonUniformity
wavelet-LHH_glszm_GrayLevelNonUniformityNormalized
wavelet-LHH_glszm_GrayLevelVariance
wavelet-LHH_glszm_HighGrayLevelZoneEmphasis
wavelet-LHH_glszm_LargeAreaEmphasis
wavelet-LHH_glszm_LargeAreaHighGrayLevelEmphasis
wavelet-LHH_glszm_LargeAreaLowGrayLevelEmphasis
wavelet-LHH_glszm_LowGrayLevelZoneEmphasis
wavelet-LHH_glszm_SizeZoneNonUniformity
wavelet-LHH_glszm_SizeZoneNonUniformityNormalized
wavelet-LHH_glszm_SmallAreaEmphasis
wavelet-LHH_glszm_SmallAreaHighGrayLevelEmphasis

wavelet-LHH_glszm_SmallAreaLowGrayLevelEmphasis
wavelet-LHH_glszm_ZoneEntropy
wavelet-LHH_glszm_ZonePercentage
wavelet-LHH_glszm_ZoneVariance
wavelet-LHH_ngtdm_Busyness
wavelet-LHH_ngtdm_Coarseness
wavelet-LHH_ngtdm_Complexity
wavelet-LHH_ngtdm_Contrast
wavelet-LHH_ngtdm_Strength
wavelet-HLL_firstorder_10Percentile
wavelet-HLL_firstorder_90Percentile
wavelet-HLL_firstorder_Energy
wavelet-HLL_firstorder_Entropy
wavelet-HLL_firstorder_InterquartileRange
wavelet-HLL_firstorder_Kurtosis
wavelet-HLL_firstorder_Maximum
wavelet-HLL_firstorder_MeanAbsoluteDeviation
wavelet-HLL_firstorder_Mean
wavelet-HLL_firstorder_Median
wavelet-HLL_firstorder_Minimum
wavelet-HLL_firstorder_Range
wavelet-HLL_firstorder_RobustMeanAbsoluteDeviation
wavelet-HLL_firstorder_RootMeanSquared
wavelet-HLL_firstorder_Skewness
wavelet-HLL_firstorder_TotalEnergy
wavelet-HLL_firstorder_Uniformity
wavelet-HLL_firstorder_Variance
wavelet-HLL_glcm_Autocorrelation
wavelet-HLL_glcm_ClusterProminence
wavelet-HLL_glcm_ClusterShade
wavelet-HLL_glcm_ClusterTendency
wavelet-HLL_glcm_Contrast
wavelet-HLL_glcm_Correlation
wavelet-HLL_glcm_DifferenceAverage
wavelet-HLL_glcm_DifferenceEntropy
wavelet-HLL_glcm_DifferenceVariance
wavelet-HLL_glcm_Id
wavelet-HLL_glcm_Idm
wavelet-HLL_glcm_Idmn
wavelet-HLL_glcm_Idn
wavelet-HLL_glcm_Imc1
wavelet-HLL_glcm_Imc2
wavelet-HLL_glcm_InverseVariance
wavelet-HLL_glcm_JointAverage
wavelet-HLL_glcm_JointEnergy
wavelet-HLL_glcm_JointEntropy
wavelet-HLL_glcm_MCC

wavelet-HLL_glcm_MaximumProbability
wavelet-HLL_glcm_SumAverage
wavelet-HLL_glcm_SumEntropy
wavelet-HLL_glcm_SumSquares
wavelet-HLL_gldm_DependenceEntropy
wavelet-HLL_gldm_DependenceNonUniformity
wavelet-HLL_gldm_DependenceNonUniformityNormalized
wavelet-HLL_gldm_DependenceVariance
wavelet-HLL_gldm_GrayLevelNonUniformity
wavelet-HLL_gldm_GrayLevelVariance
wavelet-HLL_gldm_HighGrayLevelEmphasis
wavelet-HLL_gldm_LargeDependenceEmphasis
wavelet-HLL_gldm_LargeDependenceHighGrayLevelEmphasis
wavelet-HLL_gldm_LargeDependenceLowGrayLevelEmphasis
wavelet-HLL_gldm_LowGrayLevelEmphasis
wavelet-HLL_gldm_SmallDependenceEmphasis
wavelet-HLL_gldm_SmallDependenceHighGrayLevelEmphasis
wavelet-HLL_gldm_SmallDependenceLowGrayLevelEmphasis
wavelet-HLL_glrlm_GrayLevelNonUniformity
wavelet-HLL_glrlm_GrayLevelNonUniformityNormalized
wavelet-HLL_glrlm_GrayLevelVariance
wavelet-HLL_glrlm_HighGrayLevelRunEmphasis
wavelet-HLL_glrlm_LongRunEmphasis
wavelet-HLL_glrlm_LongRunHighGrayLevelEmphasis
wavelet-HLL_glrlm_LongRunLowGrayLevelEmphasis
wavelet-HLL_glrlm_LowGrayLevelRunEmphasis
wavelet-HLL_glrlm_RunEntropy
wavelet-HLL_glrlm_RunLengthNonUniformity
wavelet-HLL_glrlm_RunLengthNonUniformityNormalized
wavelet-HLL_glrlm_RunPercentage
wavelet-HLL_glrlm_RunVariance
wavelet-HLL_glrlm_ShortRunEmphasis
wavelet-HLL_glrlm_ShortRunHighGrayLevelEmphasis
wavelet-HLL_glrlm_ShortRunLowGrayLevelEmphasis
wavelet-HLL_glszm_GrayLevelNonUniformity
wavelet-HLL_glszm_GrayLevelNonUniformityNormalized
wavelet-HLL_glszm_GrayLevelVariance
wavelet-HLL_glszm_HighGrayLevelZoneEmphasis
wavelet-HLL_glszm_LargeAreaEmphasis
wavelet-HLL_glszm_LargeAreaHighGrayLevelEmphasis
wavelet-HLL_glszm_LargeAreaLowGrayLevelEmphasis
wavelet-HLL_glszm_LowGrayLevelZoneEmphasis
wavelet-HLL_glszm_SizeZoneNonUniformity
wavelet-HLL_glszm_SizeZoneNonUniformityNormalized
wavelet-HLL_glszm_SmallAreaEmphasis
wavelet-HLL_glszm_SmallAreaHighGrayLevelEmphasis
wavelet-HLL_glszm_SmallAreaLowGrayLevelEmphasis

wavelet-HLL_glszm_ZoneEntropy
wavelet-HLL_glszm_ZonePercentage
wavelet-HLL_glszm_ZoneVariance
wavelet-HLL_ngtdm_Busyness
wavelet-HLL_ngtdm_Coarseness
wavelet-HLL_ngtdm_Complexity
wavelet-HLL_ngtdm_Contrast
wavelet-HLL_ngtdm_Strength
wavelet-HLH_firstorder_10Percentile
wavelet-HLH_firstorder_90Percentile
wavelet-HLH_firstorder_Energy
wavelet-HLH_firstorder_Entropy
wavelet-HLH_firstorder_InterquartileRange
wavelet-HLH_firstorder_Kurtosis
wavelet-HLH_firstorder_Maximum
wavelet-HLH_firstorder_MeanAbsoluteDeviation
wavelet-HLH_firstorder_Mean
wavelet-HLH_firstorder_Median
wavelet-HLH_firstorder_Minimum
wavelet-HLH_firstorder_Range
wavelet-HLH_firstorder_RobustMeanAbsoluteDeviation
wavelet-HLH_firstorder_RootMeanSquared
wavelet-HLH_firstorder_Skewness
wavelet-HLH_firstorder_TotalEnergy
wavelet-HLH_firstorder_Uniformity
wavelet-HLH_firstorder_Variance
wavelet-HLH_glcm_Autocorrelation
wavelet-HLH_glcm_ClusterProminence
wavelet-HLH_glcm_ClusterShade
wavelet-HLH_glcm_ClusterTendency
wavelet-HLH_glcm_Contrast
wavelet-HLH_glcm_Correlation
wavelet-HLH_glcm_DifferenceAverage
wavelet-HLH_glcm_DifferenceEntropy
wavelet-HLH_glcm_DifferenceVariance
wavelet-HLH_glcm_Id
wavelet-HLH_glcm_Idm
wavelet-HLH_glcm_Idmn
wavelet-HLH_glcm_Idn
wavelet-HLH_glcm_Imc1
wavelet-HLH_glcm_Imc2
wavelet-HLH_glcm_InverseVariance
wavelet-HLH_glcm_JointAverage
wavelet-HLH_glcm_JointEnergy
wavelet-HLH_glcm_JointEntropy
wavelet-HLH_glcm_MCC
wavelet-HLH_glcm_MaximumProbability

wavelet-HLH_glcm_SumAverage
wavelet-HLH_glcm_SumEntropy
wavelet-HLH_glcm_SumSquares
wavelet-HLH_gldm_DependenceEntropy
wavelet-HLH_gldm_DependenceNonUniformity
wavelet-HLH_gldm_DependenceNonUniformityNormalized
wavelet-HLH_gldm_DependenceVariance
wavelet-HLH_gldm_GrayLevelNonUniformity
wavelet-HLH_gldm_GrayLevelVariance
wavelet-HLH_gldm_HighGrayLevelEmphasis
wavelet-HLH_gldm_LargeDependenceEmphasis
wavelet-HLH_gldm_LargeDependenceHighGrayLevelEmphasis
wavelet-HLH_gldm_LargeDependenceLowGrayLevelEmphasis
wavelet-HLH_gldm_LowGrayLevelEmphasis
wavelet-HLH_gldm_SmallDependenceEmphasis
wavelet-HLH_gldm_SmallDependenceHighGrayLevelEmphasis
wavelet-HLH_gldm_SmallDependenceLowGrayLevelEmphasis
wavelet-HLH_glrlm_GrayLevelNonUniformity
wavelet-HLH_glrlm_GrayLevelNonUniformityNormalized
wavelet-HLH_glrlm_GrayLevelVariance
wavelet-HLH_glrlm_HighGrayLevelRunEmphasis
wavelet-HLH_glrlm_LongRunEmphasis
wavelet-HLH_glrlm_LongRunHighGrayLevelEmphasis
wavelet-HLH_glrlm_LongRunLowGrayLevelEmphasis
wavelet-HLH_glrlm_LowGrayLevelRunEmphasis
wavelet-HLH_glrlm_RunEntropy
wavelet-HLH_glrlm_RunLengthNonUniformity
wavelet-HLH_glrlm_RunLengthNonUniformityNormalized
wavelet-HLH_glrlm_RunPercentage
wavelet-HLH_glrlm_RunVariance
wavelet-HLH_glrlm_ShortRunEmphasis
wavelet-HLH_glrlm_ShortRunHighGrayLevelEmphasis
wavelet-HLH_glrlm_ShortRunLowGrayLevelEmphasis
wavelet-HLH_glszm_GrayLevelNonUniformity
wavelet-HLH_glszm_GrayLevelNonUniformityNormalized
wavelet-HLH_glszm_GrayLevelVariance
wavelet-HLH_glszm_HighGrayLevelZoneEmphasis
wavelet-HLH_glszm_LargeAreaEmphasis
wavelet-HLH_glszm_LargeAreaHighGrayLevelEmphasis
wavelet-HLH_glszm_LargeAreaLowGrayLevelEmphasis
wavelet-HLH_glszm_LowGrayLevelZoneEmphasis
wavelet-HLH_glszm_SizeZoneNonUniformity
wavelet-HLH_glszm_SizeZoneNonUniformityNormalized
wavelet-HLH_glszm_SmallAreaEmphasis
wavelet-HLH_glszm_SmallAreaHighGrayLevelEmphasis
wavelet-HLH_glszm_SmallAreaLowGrayLevelEmphasis
wavelet-HLH_glszm_ZoneEntropy

wavelet-HLH_glszm_ZonePercentage
wavelet-HLH_glszm_ZoneVariance
wavelet-HLH_ngtdm_Busyness
wavelet-HLH_ngtdm_Coarseness
wavelet-HLH_ngtdm_Complexity
wavelet-HLH_ngtdm_Contrast
wavelet-HLH_ngtdm_Strength
wavelet-HHL_firstorder_10Percentile
wavelet-HHL_firstorder_90Percentile
wavelet-HHL_firstorder_Energy
wavelet-HHL_firstorder_Entropy
wavelet-HHL_firstorder_InterquartileRange
wavelet-HHL_firstorder_Kurtosis
wavelet-HHL_firstorder_Maximum
wavelet-HHL_firstorder_MeanAbsoluteDeviation
wavelet-HHL_firstorder_Mean
wavelet-HHL_firstorder_Median
wavelet-HHL_firstorder_Minimum
wavelet-HHL_firstorder_Range
wavelet-HHL_firstorder_RobustMeanAbsoluteDeviation
wavelet-HHL_firstorder_RootMeanSquared
wavelet-HHL_firstorder_Skewness
wavelet-HHL_firstorder_TotalEnergy
wavelet-HHL_firstorder_Uniformity
wavelet-HHL_firstorder_Variance
wavelet-HHL_glcm_Autocorrelation
wavelet-HHL_glcm_ClusterProminence
wavelet-HHL_glcm_ClusterShade
wavelet-HHL_glcm_ClusterTendency
wavelet-HHL_glcm_Contrast
wavelet-HHL_glcm_Correlation
wavelet-HHL_glcm_DifferenceAverage
wavelet-HHL_glcm_DifferenceEntropy
wavelet-HHL_glcm_DifferenceVariance
wavelet-HHL_glcm_Id
wavelet-HHL_glcm_Idm
wavelet-HHL_glcm_Idmn
wavelet-HHL_glcm_Idn
wavelet-HHL_glcm_Imc1
wavelet-HHL_glcm_Imc2
wavelet-HHL_glcm_InverseVariance
wavelet-HHL_glcm_JointAverage
wavelet-HHL_glcm_JointEnergy
wavelet-HHL_glcm_JointEntropy
wavelet-HHL_glcm_MCC
wavelet-HHL_glcm_MaximumProbability
wavelet-HHL_glcm_SumAverage

wavelet-HHL_glcm_SumEntropy
wavelet-HHL_glcm_SumSquares
wavelet-HHL_gldm_DependenceEntropy
wavelet-HHL_gldm_DependenceNonUniformity
wavelet-HHL_gldm_DependenceNonUniformityNormalized
wavelet-HHL_gldm_DependenceVariance
wavelet-HHL_gldm_GrayLevelNonUniformity
wavelet-HHL_gldm_GrayLevelVariance
wavelet-HHL_gldm_HighGrayLevelEmphasis
wavelet-HHL_gldm_LargeDependenceEmphasis
wavelet-HHL_gldm_LargeDependenceHighGrayLevelEmphasis
wavelet-HHL_gldm_LargeDependenceLowGrayLevelEmphasis
wavelet-HHL_gldm_LowGrayLevelEmphasis
wavelet-HHL_gldm_SmallDependenceEmphasis
wavelet-HHL_gldm_SmallDependenceHighGrayLevelEmphasis
wavelet-HHL_gldm_SmallDependenceLowGrayLevelEmphasis
wavelet-HHL_glrlm_GrayLevelNonUniformity
wavelet-HHL_glrlm_GrayLevelNonUniformityNormalized
wavelet-HHL_glrlm_GrayLevelVariance
wavelet-HHL_glrlm_HighGrayLevelRunEmphasis
wavelet-HHL_glrlm_LongRunEmphasis
wavelet-HHL_glrlm_LongRunHighGrayLevelEmphasis
wavelet-HHL_glrlm_LongRunLowGrayLevelEmphasis
wavelet-HHL_glrlm_LowGrayLevelRunEmphasis
wavelet-HHL_glrlm_RunEntropy
wavelet-HHL_glrlm_RunLengthNonUniformity
wavelet-HHL_glrlm_RunLengthNonUniformityNormalized
wavelet-HHL_glrlm_RunPercentage
wavelet-HHL_glrlm_RunVariance
wavelet-HHL_glrlm_ShortRunEmphasis
wavelet-HHL_glrlm_ShortRunHighGrayLevelEmphasis
wavelet-HHL_glrlm_ShortRunLowGrayLevelEmphasis
wavelet-HHL_glszm_GrayLevelNonUniformity
wavelet-HHL_glszm_GrayLevelNonUniformityNormalized
wavelet-HHL_glszm_GrayLevelVariance
wavelet-HHL_glszm_HighGrayLevelZoneEmphasis
wavelet-HHL_glszm_LargeAreaEmphasis
wavelet-HHL_glszm_LargeAreaHighGrayLevelEmphasis
wavelet-HHL_glszm_LargeAreaLowGrayLevelEmphasis
wavelet-HHL_glszm_LowGrayLevelZoneEmphasis
wavelet-HHL_glszm_SizeZoneNonUniformity
wavelet-HHL_glszm_SizeZoneNonUniformityNormalized
wavelet-HHL_glszm_SmallAreaEmphasis
wavelet-HHL_glszm_SmallAreaHighGrayLevelEmphasis
wavelet-HHL_glszm_SmallAreaLowGrayLevelEmphasis
wavelet-HHL_glszm_ZoneEntropy
wavelet-HHL_glszm_ZonePercentage

wavelet-HHL_glszm_ZoneVariance
wavelet-HHL_ngtdm_Busyness
wavelet-HHL_ngtdm_Coarseness
wavelet-HHL_ngtdm_Complexity
wavelet-HHL_ngtdm_Contrast
wavelet-HHL_ngtdm_Strength
wavelet-HHH_firstorder_10Percentile
wavelet-HHH_firstorder_90Percentile
wavelet-HHH_firstorder_Energy
wavelet-HHH_firstorder_Entropy
wavelet-HHH_firstorder_InterquartileRange
wavelet-HHH_firstorder_Kurtosis
wavelet-HHH_firstorder_Maximum
wavelet-HHH_firstorder_MeanAbsoluteDeviation
wavelet-HHH_firstorder_Mean
wavelet-HHH_firstorder_Median
wavelet-HHH_firstorder_Minimum
wavelet-HHH_firstorder_Range
wavelet-HHH_firstorder_RobustMeanAbsoluteDeviation
wavelet-HHH_firstorder_RootMeanSquared
wavelet-HHH_firstorder_Skewness
wavelet-HHH_firstorder_TotalEnergy
wavelet-HHH_firstorder_Uniformity
wavelet-HHH_firstorder_Variance
wavelet-HHH_glcm_Autocorrelation
wavelet-HHH_glcm_ClusterProminence
wavelet-HHH_glcm_ClusterShade
wavelet-HHH_glcm_ClusterTendency
wavelet-HHH_glcm_Contrast
wavelet-HHH_glcm_Correlation
wavelet-HHH_glcm_DifferenceAverage
wavelet-HHH_glcm_DifferenceEntropy
wavelet-HHH_glcm_DifferenceVariance
wavelet-HHH_glcm_Id
wavelet-HHH_glcm_Idm
wavelet-HHH_glcm_Idmn
wavelet-HHH_glcm_Idn
wavelet-HHH_glcm_Imc1
wavelet-HHH_glcm_Imc2
wavelet-HHH_glcm_InverseVariance
wavelet-HHH_glcm_JointAverage
wavelet-HHH_glcm_JointEnergy
wavelet-HHH_glcm_JointEntropy
wavelet-HHH_glcm_MCC
wavelet-HHH_glcm_MaximumProbability
wavelet-HHH_glcm_SumAverage
wavelet-HHH_glcm_SumEntropy

wavelet-HHH_glcm_SumSquares
wavelet-HHH_gldm_DependenceEntropy
wavelet-HHH_gldm_DependenceNonUniformity
wavelet-HHH_gldm_DependenceNonUniformityNormalized
wavelet-HHH_gldm_DependenceVariance
wavelet-HHH_gldm_GrayLevelNonUniformity
wavelet-HHH_gldm_GrayLevelVariance
wavelet-HHH_gldm_HighGrayLevelEmphasis
wavelet-HHH_gldm_LargeDependenceEmphasis
wavelet-HHH_gldm_LargeDependenceHighGrayLevelEmphasis
wavelet-HHH_gldm_LargeDependenceLowGrayLevelEmphasis
wavelet-HHH_gldm_LowGrayLevelEmphasis
wavelet-HHH_gldm_SmallDependenceEmphasis
wavelet-HHH_gldm_SmallDependenceHighGrayLevelEmphasis
wavelet-HHH_gldm_SmallDependenceLowGrayLevelEmphasis
wavelet-HHH_glrlm_GrayLevelNonUniformity
wavelet-HHH_glrlm_GrayLevelNonUniformityNormalized
wavelet-HHH_glrlm_GrayLevelVariance
wavelet-HHH_glrlm_HighGrayLevelRunEmphasis
wavelet-HHH_glrlm_LongRunEmphasis
wavelet-HHH_glrlm_LongRunHighGrayLevelEmphasis
wavelet-HHH_glrlm_LongRunLowGrayLevelEmphasis
wavelet-HHH_glrlm_LowGrayLevelRunEmphasis
wavelet-HHH_glrlm_RunEntropy
wavelet-HHH_glrlm_RunLengthNonUniformity
wavelet-HHH_glrlm_RunLengthNonUniformityNormalized
wavelet-HHH_glrlm_RunPercentage
wavelet-HHH_glrlm_RunVariance
wavelet-HHH_glrlm_ShortRunEmphasis
wavelet-HHH_glrlm_ShortRunHighGrayLevelEmphasis
wavelet-HHH_glrlm_ShortRunLowGrayLevelEmphasis
wavelet-HHH_glszm_GrayLevelNonUniformity
wavelet-HHH_glszm_GrayLevelNonUniformityNormalized
wavelet-HHH_glszm_GrayLevelVariance
wavelet-HHH_glszm_HighGrayLevelZoneEmphasis
wavelet-HHH_glszm_LargeAreaEmphasis
wavelet-HHH_glszm_LargeAreaHighGrayLevelEmphasis
wavelet-HHH_glszm_LargeAreaLowGrayLevelEmphasis
wavelet-HHH_glszm_LowGrayLevelZoneEmphasis
wavelet-HHH_glszm_SizeZoneNonUniformity
wavelet-HHH_glszm_SizeZoneNonUniformityNormalized
wavelet-HHH_glszm_SmallAreaEmphasis
wavelet-HHH_glszm_SmallAreaHighGrayLevelEmphasis
wavelet-HHH_glszm_SmallAreaLowGrayLevelEmphasis
wavelet-HHH_glszm_ZoneEntropy
wavelet-HHH_glszm_ZonePercentage
wavelet-HHH_glszm_ZoneVariance

wavelet-HHH_ngtdm_Busyness
wavelet-HHH_ngtdm_Coarseness
wavelet-HHH_ngtdm_Complexity
wavelet-HHH_ngtdm_Contrast
wavelet-HHH_ngtdm_Strength
wavelet-LLL_firstorder_10Percentile
wavelet-LLL_firstorder_90Percentile
wavelet-LLL_firstorder_Energy
wavelet-LLL_firstorder_Entropy
wavelet-LLL_firstorder_InterquartileRange
wavelet-LLL_firstorder_Kurtosis
wavelet-LLL_firstorder_Maximum
wavelet-LLL_firstorder_MeanAbsoluteDeviation
wavelet-LLL_firstorder_Mean
wavelet-LLL_firstorder_Median
wavelet-LLL_firstorder_Minimum
wavelet-LLL_firstorder_Range
wavelet-LLL_firstorder_RobustMeanAbsoluteDeviation
wavelet-LLL_firstorder_RootMeanSquared
wavelet-LLL_firstorder_Skewness
wavelet-LLL_firstorder_TotalEnergy
wavelet-LLL_firstorder_Uniformity
wavelet-LLL_firstorder_Variance
wavelet-LLL_glcm_Autocorrelation
wavelet-LLL_glcm_ClusterProminence
wavelet-LLL_glcm_ClusterShade
wavelet-LLL_glcm_ClusterTendency
wavelet-LLL_glcm_Contrast
wavelet-LLL_glcm_Correlation
wavelet-LLL_glcm_DifferenceAverage
wavelet-LLL_glcm_DifferenceEntropy
wavelet-LLL_glcm_DifferenceVariance
wavelet-LLL_glcm_Id
wavelet-LLL_glcm_Idm
wavelet-LLL_glcm_Idmn
wavelet-LLL_glcm_Idn
wavelet-LLL_glcm_Imc1
wavelet-LLL_glcm_Imc2
wavelet-LLL_glcm_InverseVariance
wavelet-LLL_glcm_JointAverage
wavelet-LLL_glcm_JointEnergy
wavelet-LLL_glcm_JointEntropy
wavelet-LLL_glcm_MCC
wavelet-LLL_glcm_MaximumProbability
wavelet-LLL_glcm_SumAverage
wavelet-LLL_glcm_SumEntropy
wavelet-LLL_glcm_SumSquares

wavelet-LLL_gldm_DependenceEntropy
wavelet-LLL_gldm_DependenceNonUniformity
wavelet-LLL_gldm_DependenceNonUniformityNormalized
wavelet-LLL_gldm_DependenceVariance
wavelet-LLL_gldm_GrayLevelNonUniformity
wavelet-LLL_gldm_GrayLevelVariance
wavelet-LLL_gldm_HighGrayLevelEmphasis
wavelet-LLL_gldm_LargeDependenceEmphasis
wavelet-LLL_gldm_LargeDependenceHighGrayLevelEmphasis
wavelet-LLL_gldm_LargeDependenceLowGrayLevelEmphasis
wavelet-LLL_gldm_LowGrayLevelEmphasis
wavelet-LLL_gldm_SmallDependenceEmphasis
wavelet-LLL_gldm_SmallDependenceHighGrayLevelEmphasis
wavelet-LLL_gldm_SmallDependenceLowGrayLevelEmphasis
wavelet-LLL_glrlm_GrayLevelNonUniformity
wavelet-LLL_glrlm_GrayLevelNonUniformityNormalized
wavelet-LLL_glrlm_GrayLevelVariance
wavelet-LLL_glrlm_HighGrayLevelRunEmphasis
wavelet-LLL_glrlm_LongRunEmphasis
wavelet-LLL_glrlm_LongRunHighGrayLevelEmphasis
wavelet-LLL_glrlm_LongRunLowGrayLevelEmphasis
wavelet-LLL_glrlm_LowGrayLevelRunEmphasis
wavelet-LLL_glrlm_RunEntropy
wavelet-LLL_glrlm_RunLengthNonUniformity
wavelet-LLL_glrlm_RunLengthNonUniformityNormalized
wavelet-LLL_glrlm_RunPercentage
wavelet-LLL_glrlm_RunVariance
wavelet-LLL_glrlm_ShortRunEmphasis
wavelet-LLL_glrlm_ShortRunHighGrayLevelEmphasis
wavelet-LLL_glrlm_ShortRunLowGrayLevelEmphasis
wavelet-LLL_glszm_GrayLevelNonUniformity
wavelet-LLL_glszm_GrayLevelNonUniformityNormalized
wavelet-LLL_glszm_GrayLevelVariance
wavelet-LLL_glszm_HighGrayLevelZoneEmphasis
wavelet-LLL_glszm_LargeAreaEmphasis
wavelet-LLL_glszm_LargeAreaHighGrayLevelEmphasis
wavelet-LLL_glszm_LargeAreaLowGrayLevelEmphasis
wavelet-LLL_glszm_LowGrayLevelZoneEmphasis
wavelet-LLL_glszm_SizeZoneNonUniformity
wavelet-LLL_glszm_SizeZoneNonUniformityNormalized
wavelet-LLL_glszm_SmallAreaEmphasis
wavelet-LLL_glszm_SmallAreaHighGrayLevelEmphasis
wavelet-LLL_glszm_SmallAreaLowGrayLevelEmphasis
wavelet-LLL_glszm_ZoneEntropy
wavelet-LLL_glszm_ZonePercentage
wavelet-LLL_glszm_ZoneVariance
wavelet-LLL_ngtdm_Busyness

wavelet-LLL_ngtdm_Coarseness
wavelet-LLL_ngtdm_Complexity
wavelet-LLL_ngtdm_Contrast
wavelet-LLL_ngtdm_Strength
